# Surfaceome Profiling of Cell Lines and Patient-Derived Xenografts Confirm FGFR4, NCAM1, CD276, and Highlight AGRL2, JAM3, and L1CAM as Surface Targets for Rhabdomyosarcoma

**DOI:** 10.3390/ijms24032601

**Published:** 2023-01-30

**Authors:** Andrea Timpanaro, Caroline Piccand, Anne-Christine Uldry, Peter Karl Bode, Dzhangar Dzhumashev, Rita Sala, Manfred Heller, Jochen Rössler, Michele Bernasconi

**Affiliations:** 1 Department of Pediatric Hematology and Oncology, Inselspital, Bern University Hospital, University of Bern, 3010 Bern, Switzerland; 2 Translational Cancer Research, Department for BioMedical Research (DBMR), University of Bern, 3008 Bern, Switzerland; 3 Graduate School for Cellular and Biomedical Sciences, University of Bern, 3012 Bern, Switzerland; 4 Proteomics & Mass Spectrometry Core Facility, Department for BioMedical Research (DBMR), University of Bern, 3008 Bern, Switzerland; 5 Department of Pathology and Molecular Pathology, University Hospital Zurich, 8091 Zurich, Switzerland; 6 Children’s Research Center, University Children’s Hospital Zurich, University of Zurich, 3032 Zurich, Switzerland

**Keywords:** rhabdomyosarcoma, cell surface proteomics, targeted therapies, antibody-based therapies, AGRL2, JAM3, L1CAM

## Abstract

Rhabdomyosarcoma (RMS) is the most common soft tissue sarcoma in children. The prognosis for patients with high-grade and metastatic disease is still very poor, and survivors are burdened with long-lasting side effects. Therefore, more effective and less toxic therapies are needed. Surface proteins are ideal targets for antibody-based therapies, like bispecific antibodies, antibody-drug conjugates, or chimeric antigen receptor (CAR) T-cells. Specific surface targets for RMS are scarce. Here, we performed a surfaceome profiling based on differential centrifugation enrichment of surface/membrane proteins and detection by LC-MS on six fusion-positive (FP) RMS cell lines, five fusion-negative (FN) RMS cell lines, and three RMS patient-derived xenografts (PDXs). A total of 699 proteins were detected in the three RMS groups. Ranking based on expression levels and comparison to expression in normal MRC-5 fibroblasts and myoblasts, followed by statistical analysis, highlighted known RMS targets such as FGFR4, NCAM1, and CD276/B7-H3, and revealed AGRL2, JAM3, MEGF10, GPC4, CADM2, as potential targets for immunotherapies of RMS. L1CAM expression was investigated in RMS tissues, and strong L1CAM expression was observed in more than 80% of alveolar RMS tumors, making it a practicable target for antibody-based therapies of alveolar RMS.

## 1. Introduction

Pediatric rhabdomyosarcoma (RMS) is the most common soft tissue sarcoma in children and young adults [1]. Each year it accounts for 3% of childhood cancers in the United States [2]. RMS is a heterogeneous group of malignant and metastatic tumors, which originate from a primitive mesenchymal cell [3]. Based on histology, RMS can be classified into different subtypes: embryonal RMS (eRMS; 60–70%) and alveolar RMS (aRMS; 20–30%) are the main subtypes; pleomorphic (pRMS) and spindle cell/sclerosing (s-scRMS) account for 7–15% of the cases [4]. The aggressive aRMS tumors carry one of the two characteristic chromosomal translocations, the t(2; 13)(q35; q14) or the t(1; 13)(p36; q14), which result in the expression of a PAX3-FOXO1 or PAX7-FOXO1 fusion transcription factor, respectively [5], and are therefore also called fusion-positive (FP). Embryonal RMS tumors have a better prognosis and are clinically indistinguishable from fusion-negative (FN) RMS [6].

Although overall five-year survival rates have improved with the combined use of surgery, radiation therapy and chemotherapy, prognosis remains poor in pediatric patients with metastatic and aggressive diseases, such as aRMS [7,8]. Moreover, long-term toxicities of the intense chemotherapy/radiation therapy regimens are now becoming more evident with improving survival [9,10]. Therefore, new therapies are desperately needed for children and young adults with high-risk and recurrent solid tumors.

Cell surface proteins are ideal targets for antibody-based therapies, like antibody-drug conjugates [11,12], bispecific antibodies [13,14], or chimeric antigen receptor (CAR) T cells [15,16,17]. However, specific surface antigens for RMS are scarce. Before the advent of omics technologies, the study of muscle development and biology revealed several surface proteins upregulated in RMS tumors, including the Insulin-like Growth Factor Receptor 1 (IGF1R/CD221) [18,19,20], the gamma subunit of the Acetylcholine Receptor (ACHG) [21], the Neural Cell Adhesion Molecule 1 (NCAM1/CD56) [22,23,24,25], and the Receptor tyrosine-protein kinase erbB-2 (ERBB2/HER2) [26,27,28,29,30]. Antibodies against IGFR1 were active in preclinical models [31,32], but failed to meet the expected clinical outcome. They achieved partial remissions in Ewing Sarcoma and RMS patients, but development and production were halted [33]. ACHG followed the same fate, despite having an extremely favorable preferential expression profile in RMS. Monoclonal antibodies and CAR T-cells have failed to achieve a significant preclinical effect [34], possibly because of the low copy numbers of the receptor on the surface. An antibody-drug conjugate (ADC) targeting NCAM1/CD56 was well tolerated in children, including RMS patients, but has shown limited clinical activity so far [35]. On the bright side, CAR T-cells targeting HER2 have promisingly contributed to inducing remission in a patient with metastatic RMS [36].

The advent of microarrays, and later of next-generation RNA sequencing led to the identification of several genes coding for surface proteins highly expressed in RMS tumors, *FGFR4* being the most prominent [37,38,39]. Monoclonal antibodies [40], ADC [41], and CAR T cells are being developed with promising preclinical results [42,43]. Gene expression profiling has revealed that the gene coding for the cannabinoid receptor 1 (*CB1*) is highly upregulated in FP-RMS [44], but no targeting approaches have been developed yet. With more and more datasets available, efforts dedicated to identifying surface proteins have led to the identification of potential immunotherapy targets for pediatric solid tumors [45,46]. Specific targets identified for RMS included FGFR4, GPC3 and GPC5, and FOLR1 (alpha-folate receptor). Other important studies have been conducted to unravel the genomics [47] and (phospho)proteomics [48] profiles of RMS, but these efforts were not focused on defining the surface proteins expressed by RMS.

Recently, the immuno-transcriptomic profiling of a large set of extracranial pediatric solid tumors, including 129 RMS tumors and 35 RMS cell lines, defined the level of expression of the genes coding for surface proteins, providing an extremely useful resource to identify and evaluate potential targets [49]. Nevertheless, the complex relationship between mRNA and protein abundance, influenced by post-transcriptional and translational mechanisms, as well as by protein degradation [50], makes it hard to select specific surface targets from transcriptomics analysis.

So far, only one study dedicated to the identification of surface proteins in RMS has been reported [51]. This study, performed with two FP-RMS, two FN-RMS cell lines, and one RMS PDX, identified several surface proteins expressed in RMS, highlighting a possible role of B7-H3/CD276 in immune evasion.

Here, we report the surfaceome profiling of six FP-RMS and five FN-RMS cell lines and three PDXs, by differential centrifugation enrichment of surface/membrane proteins and mass spectrometry (MS) analysis, leading to the identification of 699 proteins expressed in RMS and of three novel putative cell surface targets for immunotherapy of RMS.

## 2. Results

### 2.1. Isolation and Enrichment of Membrane/Surface Proteins

**Figure 1:**
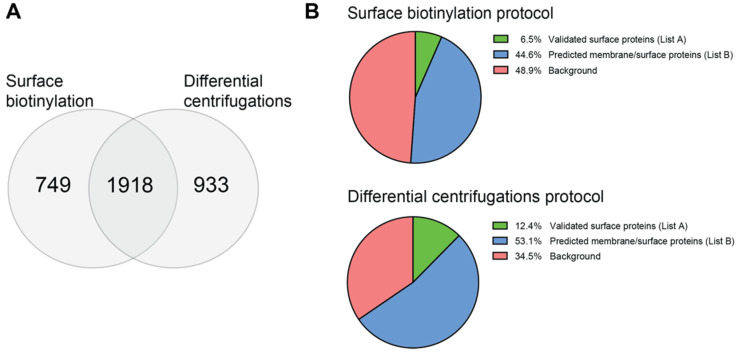
Comparison of surface biotinylation and differential centrifugations for the enrichment of membrane/surface proteins. Two methods for the enrichment of membrane/surface proteins were compared in a pilot experiment with the Rh4 cell line in triplicates. (**A**) More proteins were detected after differential centrifugations enrichment than after surface biotinylation, but there was a consistent overlap between the two methods. (**B**) Differential centrifugations resulted in the enrichment of a higher number of membrane/surface proteins than surface biotinylation and in a lower background of intracellular proteins. Created with Biorender.com.

In order to identify novel and specific targets upregulated on the surface of rhabdomyosarcoma (RMS) cells by mass spectrometry (MS), we initially compared two methods for isolation of membrane/surface proteins: the first based on biotin labeling of cell surface proteins with the cleavable EZ-Link-Sulfo-NHS-SS-biotin, followed by isolation with a NeutrAvidin agarose column, and reducing elution with dithiothreitol (DTT); the second based on differential centrifugations and washes at high pH and high salts concentration [52]. In a preliminary experiment performed in triplicates with the Rh4 cell line, we could detect MS 2667 proteins with the surface biotinylation method and 2851 proteins with the differential centrifugations method. A total of 1918 proteins were detected with both methods (Figure 1A). A low enrichment for surface proteins and the high sensitivity of MS may often result in the detection of intracellular proteins. 

To determine the enrichment efficiency of the two methods, the detected proteins were filtered with a list of 2886 annotated surface proteins, published by Bausch-Fluck et al. [53] (Supplementary Information List A), and with a list containing 7643 proteins compiled to include all the annotated membrane/surface proteins (Supplementary Information List B). This analysis showed that the differential centrifugations protocol produced a lower background (~35%) compared to the biotinylation protocol, which resulted in the detection of ~49% of intracellular proteins (Figure 1B). Interestingly, the differential centrifugations protocol resulted in a higher enrichment of annotated and predicted surface proteins. Therefore, this method was used for the next experiments.

### 2.2. Surfaceome Profiling Strategy and Proteomics Results Analysis

**Figure 2:**
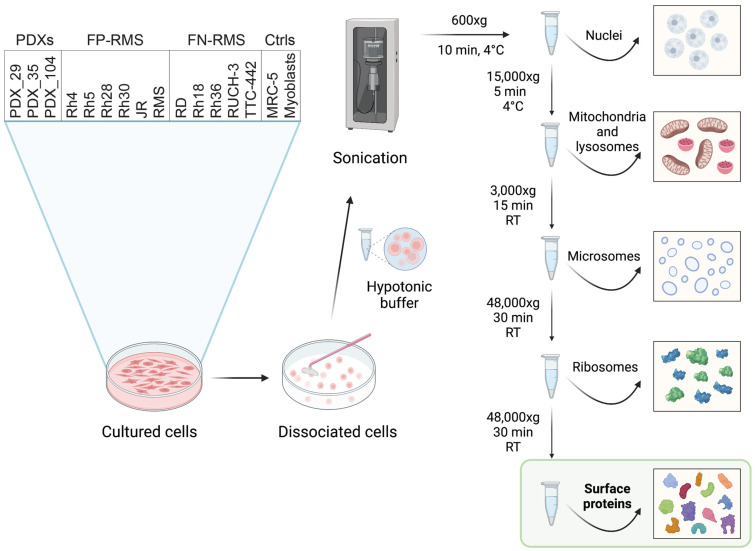
RMS cell lines and PDXs are used for differential centrifugation enrichment of membrane/surface proteins. RMS cell lines and PDXs, as well as the normal controls MRC-5 human embryonal fibroblasts and primary human myoblast, were cultured and used for the isolation of membrane/surface proteins. All experiments were performed in triplicates. Created with Biorender.com.

Eleven RMS cell lines, three PDXs, and MRC-5 human embryonal fibroblasts and primary myoblasts, as controls, were cultured, and surface membrane proteins were enriched following the differential centrifugations protocol, as shown in Figure 2.

**Figure 3:**
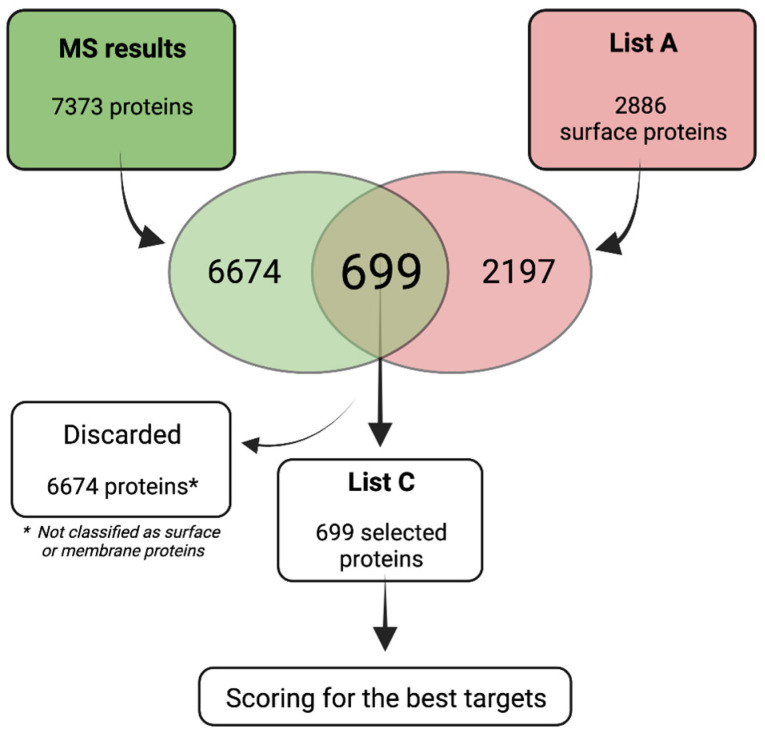
Schematic outline of the strategy used to analyze mass spectrometry data. The surfaceome of six FP-RMS cell lines, five FN-RMS cell lines, and three RMS PDXs were analyzed by MS. The strategy adopted to analyze the MS data is shown. The MS results were filtered with a list of annotated surface proteins (List A). A total of 699 proteins predicted to be surface proteins (List C) were then further prioritized by a scoring strategy to identify highly expressed proteins specific to RMS.

The isolated proteins were then processed, and detection was performed by LC-MS. In total, 7373 proteins were detected and quantified (iTop3 values in Supplementary Information Tables S1 and S2). To analyze the MS data, we then applied the strategy summarized in Figure 3. 

The 7373 proteins were then filtered with List A, revealing 699 membrane/surface proteins that were then selected to generate List C (Supplementary Information Table S3. To prioritize membrane/surface proteins with high and consistent expression in RMS cells lines and PDXs, and low or absent expression in controls, List C was processed with a scoring strategy taking into account the following parameters: (1) a number of RMS cell lines in which a protein was detected; (2) abundance mean, defined as “iTop3 mean”, of all the RMS cell lines; (3) ratio of the iTop3 values between PDXs, FP-RMS, and FN-RMS, with the controls MRC-5 and the primary myoblasts, expressed as the base-2 logarithm of Fold Change (Log_2_(FC)); (4) no detection in the controls MRC-5 and primary myoblasts; (5) high expression in the PDXs, since these are biologically closer to primary tumors (Table 1 and Table 2).

This approach attributes the lowest scores to the most abundant proteins in the three groups, PDXs, FP-RMS and FN-RMS, but not in the controls. An analysis of proteins upregulated two-fold in the different groups is available in Supplementary Information Tables S4–S7. The comprehensive list of all the ranked proteins is available as Supplementary Information (Supplementary Information Table S8). The first 100 proteins ranked by this scoring are presented in Table A1, as Top100, and in detail in Supplementary Information Tables S9 and S10.

### 2.3. Statistical Analysis of the Filtered Proteins Highlights Five Putative Therapeutic RMS Surface Targets

In parallel to the above selection of surface proteins, two different statistical analyses were performed in order to identify the most significant putative surface targets.

First, an individual cell-to-control differential expression test was performed. More specifically, the Empirical Bayes (EB) or moderated *t*-test was applied, as implemented in R [54,55].

**Figure 4:**
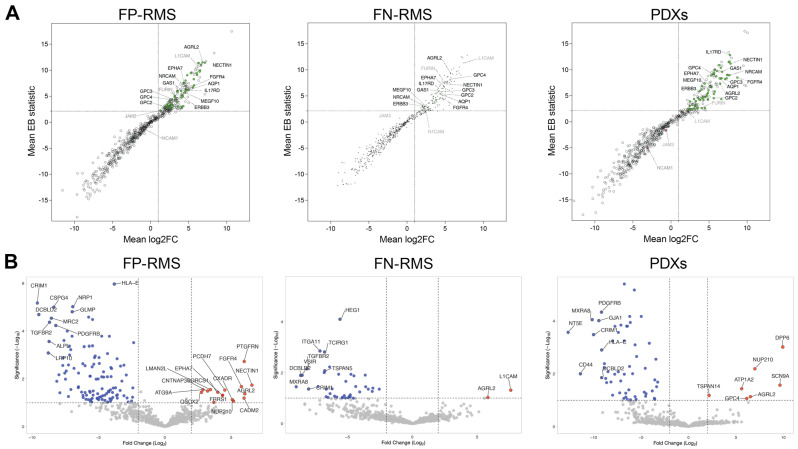
AGRL2 is significantly upregulated in FP-RMS, FN-RMS cell lines, and RMS PDXs. Results for the 699 proteins of interest (List C) using the imputed iTop3 intensities are shown for each set of cells. Two statistical analyses were used: (**A**) Cell-to-control differential expression. Shown here are the average log_2_ fold changes between each cell of the set and MRC-5 and myoblasts versus the average of the corresponding moderated t-statistics of the comparisons. The proteins for which the moderated t-statistics were above 2.132 (95th percentile of the corresponding Student’s distribution) in all three sets of cells are highlighted in green, with some of the proteins present in the Top100 labeled. (**B**) Linear mixed model (LMM) was used as a statistical evaluation of the protein abundances detected in all the distinct groups (FP-RMS, FN-RMS, and PDXs) versus the controls. A threshold of significance (−log_10_) ≥ 1.3 and|log_2_FC| ≥ 1 was set to plot the statistically significant proteins in volcano plots. AGRL2 is the only protein significantly upregulated in all three RMS groups with this analysis.

Considering an average Log_2_(FoldChange) ≥ 2 versus an average EB statistic ≥ 2.132 across comparisons within a class (FP-RMS, FN-RMS, and PDXs), 63 proteins were identified as upregulated in all RMS groups, 32 of which were present in the Top100 corroborating our first selection (Supplementary Table S14). Among these, AGRL2, AQP1, EPHA7, ERBB3, FGFR4, GAS1, GPC2, GPC3, GPC4, IL17RD, MEGF10, NRCAM, NECTIN1 are highlighted in Figure 4A. L1CAM was significantly upregulated in FP-RMS and FN-RMS, NCAM1 only in FN-RMS, and JAM-3 only in FP-RMS (Figure 4A). 

The second statistical analysis, called linear mixed model (LMM) and derived from the R implementation DREAM [56], is a statistical evaluation of all the respective FP-RMS, FN-RMS, and PDXs groups versus the controls, even though the groups are themselves collection of subgroups of replicates. The LMM analysis considers the variations within the cell lines as well. The LMM results were very stringent, and only AGRL2 was confirmed as significantly overexpressed in all three RMS groups. FGFR4 was identified in FP-RMS, L1CAM in FN-RMS, and GPC4 in PDXs (Figure 4B and Supplementary Information Table S13). To note is that LMM selected a larger number of downregulated than upregulated proteins in the RMS groups compared to the controls.

In conclusion, extended statistical analyses detected AGRL2, ranked first by our ranking strategy, as significantly overexpressed in all samples. Detection in several groups of FGFR4, a well-established target for RMS, validates our approach.

### 2.4. Expression of the Top100 Proteins in Normal Tissues

**Figure 5:**
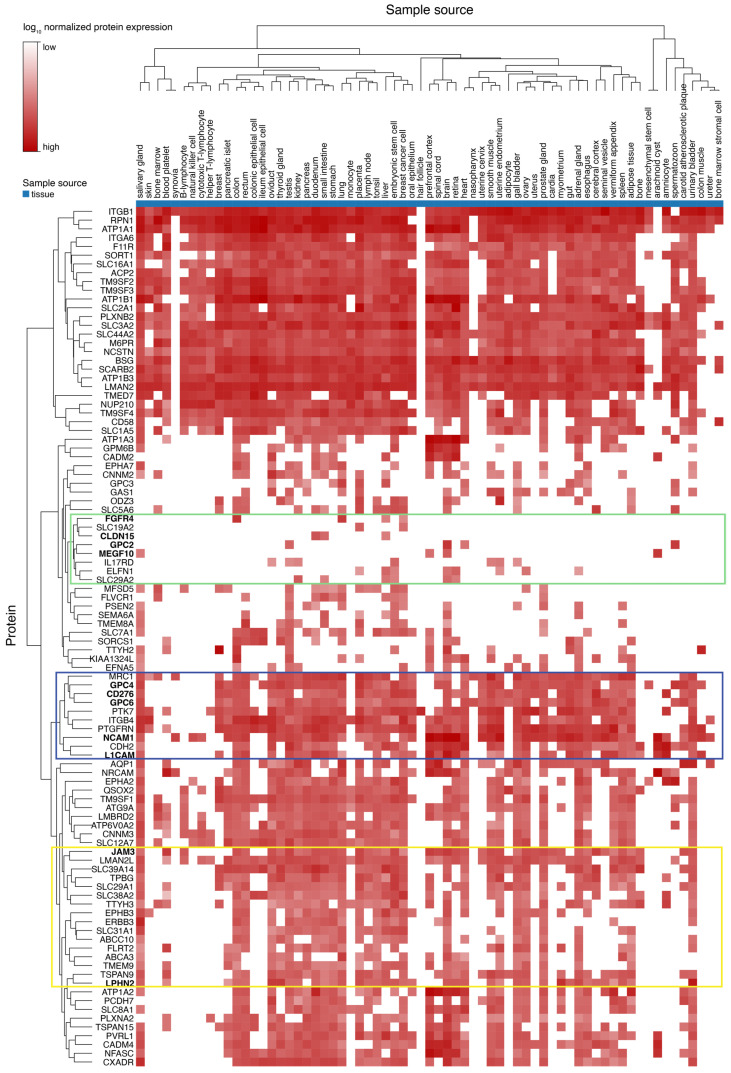
Top100 proteins expression in normal tissues. Protein expression levels expressed as normalized iTop3 values for the selected Top100 proteins in normal tissues were generated with the ProteomicsDB database analytic toolbox expression heat map by selecting Tissue, MS1, Top3, and SWISS-PROT only. FGFR4 and GPC2 (green square) were detected in a few normal tissues. GPC4, CD276, N1CAM, and L1CAM (blue square) cluster close together and are expressed at low-medium levels in most of the considered normal tissues. AGRL2 (indicated as LPHN2 in the figure) and JAM3 (yellow square) cluster loosely together and are detected in several normal tissues but mostly at low levels. The values represented can be found in Supplementary Information Table S15, and are the mean total sum normalized protein expression value across all samples that are stored in the database PrtoteomicsDB.

During the selection of the putative targets, we considered MRC-5 normal embryonal fibroblasts and immortalized primary myoblasts as controls. An ideal RMS target should be expressed at high levels in RMS and not, or at low levels, in all normal tissues. Therefore, to evaluate the expression of the Top100 proteins in normal tissues, we took advantage of proteomics data for normal tissues available from Proteomicsdb.org (accessed on 25 November 2022) [57,58,59]. The expression heatmap generated with the MS1 Top3 values (Tissue, SWISS-PROT only) confirms that FGFR4 is a very specific target because it is detected only in the colon, lung, and liver (Figure 5, green square) and highlights other excellent targets clustering together with FGFR4: Glypican-2 (GPC2), detected only in the testis and heart at low levels, and in spermatozoon and brain at medium levels; Multiple epidermal growth factor-like domains protein 10 (MEGF10), detected in brain, prefrontal cortex, and salivary gland at low levels, and in arachnoid cyst at medium levels; and Claudin-15 (CLDN15), detected only in duodenum, liver, and small intestine at low levels (Figure 5, green square). 

Moreover, a cluster of candidates (Figure 5, blue square), including GPC4, GPC6, CD276, NCAM1, and L1CAM, are detected only at low-medium levels in most tissues. Interestingly, AGRL2 (LPHN2) and JAM3 (Figure 5, yellow square) clustering loosely together are detected in about 30 tissues but at low levels in almost all of them. For AGRL2, the highest expression is detected in the urinary bladder, myometrium, thyroid gland, oviduct, adrenal gland, and placenta.

### 2.5. Specific and High mRNA Expression of the Candidates in Patients’ RMS Samples

**Figure 6:**
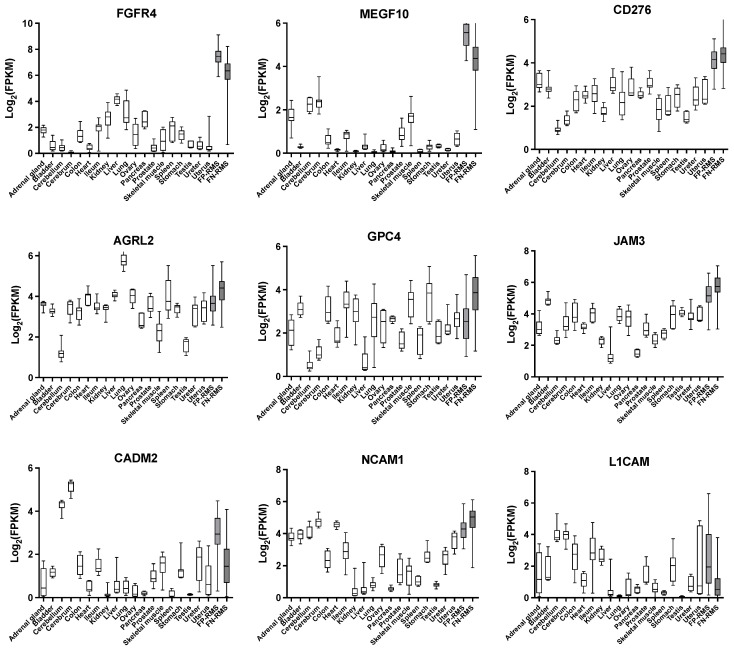
Transcript expression of selected candidates in RMS patients’ samples and normal tissues. Transcript numbers were obtained from a published RNAseq data set of 38 FP-RMS, 60 FN-RMS, and 5–20 normal tissues per organ [49]. *FGFR4*, *MEGF10*, and *CD276* show a clear higher expression in tumors (grey boxes) compared to normal tissues (white boxed). Box and whiskers show the median with the 25th to 75th percentiles. Bars represent the minimum and maximum values.

Since a direct comparison of our data with the normalized proteomic expression data in normal tissues from ProteomicsDB (Figure 5) is not possible, to investigate the therapeutic potential of the selected targets, we analyzed their expression in RMS patients’ samples and in normal tissues, by using transcriptomics data published by Brohl et al. [49] (Supplementary Information Tables S11 and S12). Transcriptomics analysis of normal tissues confirms the selective RMS expression of the candidates. Indeed, the most representative candidates, e.g., *FGFR4*, show a relatively low FPKM number in normal tissues when compared to RMS tumors, where expression is highest in FP-RMS (Figure 6). Highly specific expression of *MEGF10* was observed in tumor samples, particularly in FP-RMS, compared to normal tissues. For *MEGF10*, the highest expression among normal tissues is observed in the cerebrum and cerebellum. *CD276*, *JAM3*, and *NCAM1* also show higher expression in tumors compared to normal tissues, although expression in normal tissues is higher than for *FGFR4* and *MEGF10*. Expression of *GPC4* is high in the lungs, of *L1CAM* in the brain, and of GPC4 in the stomach. For these targets, a careful evaluation of protein expression in normal tissues will be required.

**Figure 7:**
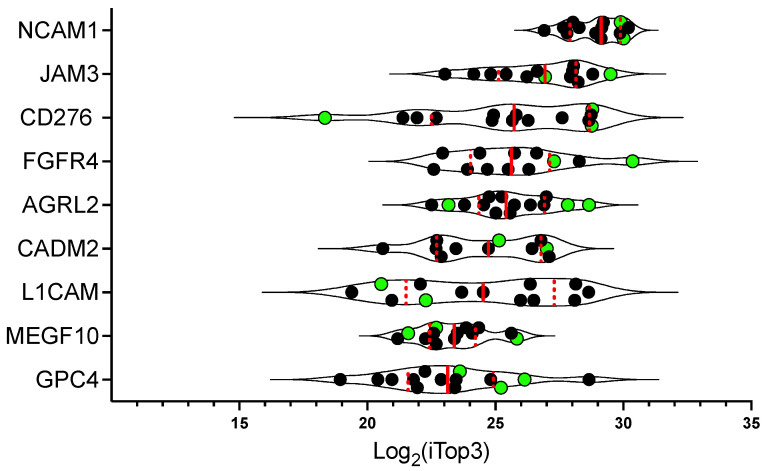
Proteomics abundance distribution of the candidates in the RMS samples. Log_2_(iTop3) of all the samples were plotted for the selected membrane/surface proteins. The proteins were ranked by the median expression from top to bottom. The highest expression was consistently identified in PDXs (green dots), except for L1CAM, which was expressed at lower levels in PDXs compared to cell lines. Shown are the median (red line) and quartiles (dotted red line).

Next, we analyzed the distribution of peptides abundance of the most promising putative targets with a Log_2_(FC) > 2 in the RMS samples (Figure 7). The highest median Log_2_(iTop3) value was observed for NCAM1, followed by JAM3, CD276, FGFR4, AGRL2, CADM2, L1CAM, MEGF10, and GPC4. Importantly, all were consistently found in PDXs (green dots). 

Taken together, these results validate our selection strategy and show that the targets of interest were indeed detected at highest levels on PDXs, suggesting that they might be valuable therapeutic targets for RMS.

### 2.6. Validation of AGRL2, L1CAM, and JAM3 Expression on RMS Cell Lines

**Figure 8:**
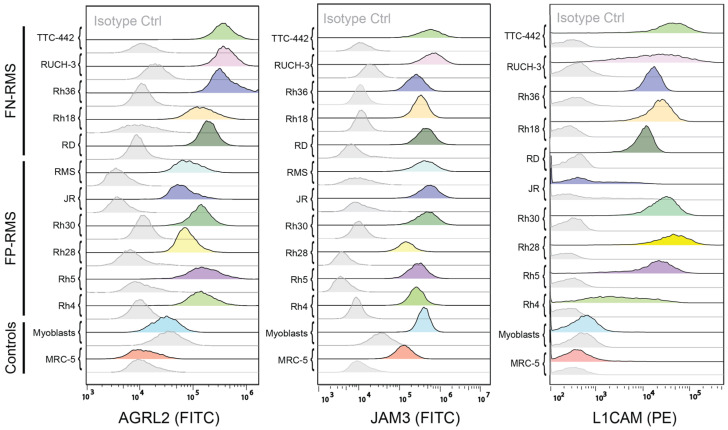
Surface expression of the selected targets on RMS cell lines. Expression of AGRL2, JAM3, and L1CAM was measured by FACS on eleven RMS cell lines, and on MRC-5 fibroblasts and myoblasts as controls. For AGRL2 and JAM3, the primary antibody was incubated with 100,000 cells and detected with an Alexa488-conjugated secondary antibody. For L1CAM, a PE-labeled antibody was used. A total of 10,000 events were collected.

After performing surfaceome analysis and in silico selection for RMS surface targets, several candidates stood out in terms of high expression in RMS samples (NCAM1, JAM3, CD276, FGFR4, AGRL2, CADM2, L1CAM, and MEGF10) and some showed a particular low expression in normal tissues (FGFR4, MEGF10, and CD276). FGFR4 and NCAM1 are known targets for RMS; therefore, to reveal novel targets for RMS, we selected AGRL2, JAM3, and L1CAM and investigated the surface expression by Flow Cytometry on the eleven RMS cell lines and the two controls, MRC-5 and myoblasts, used in this study (Figure 8). For AGRL2 and JAM3, no directly labeled antibodies are commercially available; therefore, we had to use a two-step incubation with fluorescent secondary antibodies. 

All RMS cells were positive for AGRL2, while the staining for MRC-5 fibroblasts and myoblasts was not above the control staining. The FP-RMS cell lines showed stronger staining than FN-RMS cell lines. JAM3 staining of RMS cell lines was consistently higher than AGRL2; however, staining in myoblasts and MRC-5 fibroblasts was higher than with isotype control, even though it was clearly lower than in RMS cell lines. L1CAM staining was clearly much higher in RMS cell lines compared to the controls. Therefore, these results demonstrate that the three proteins are expressed at high levels on most RMS cell lines and are expressed at much lower levels in the controls. This, on one side, validates our surfaceome profiling and selection strategy and, on the other side, reveals AGRL2, JAM3, and L1CAM as novel surface targets for RMS. 

### 2.7. Expression of L1CAM in RMS Tumors and Inverse Correlation with Survival

**Figure 9:**
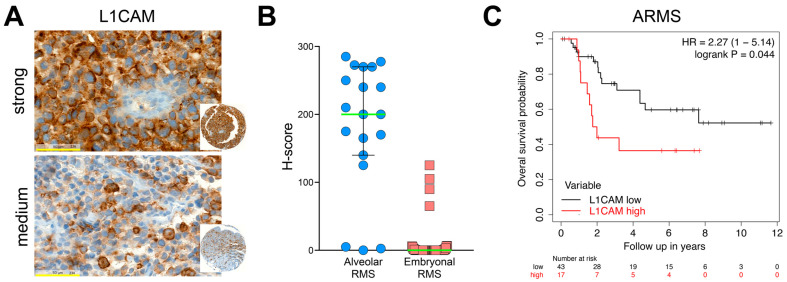
L1CAM is detected in the majority of ARMS cases by IHC, and a high L1CAM mRNA expression correlates with poorer survival. A tissue microarray (TMA) including 24 ARMS and 100 ERMS was stained with L1CAM antibody clone 14.10. (**A**) Upper panel: ARMS sample with strong L1CAM staining, A. lower panel, ARMS sample with medium L1CAM staining. Magnification 40×, yellow bars represent 50 µm. (**B**) H-score for L1CAM was calculated by adding the percentage of cells showing strong staining multiplied by 3, to medium staining by 2 and to weak staining. Evaluable tissues: ARMS *n* = 19, ERMS *n* = 60. Green bars represent the median value, with a 95% confidence interval. (**C**) Kaplan–Meier survival plot of patients with ARMS comparing high and low L1CAM mRNA expression. Data were obtained from the cohort Davicioni 147 [39] and deposited to the R2: Genomics Analysis and Visualization Platform (http://r2.amc.nl (accessed on 12 January 2023)). Analysis was performed on the ARMS subset using the custom Kaplan–Meier tool (http://kmplot.com (accessed on 14 January 2023)). Shown are the hazard rate (HR) and the Cox–Mantel (logrank) test [61].

We next investigated the expression of L1CAM on a tissue microarray (TMA) with 248 cores from 124 RMS tumors, consisting of 24 ARMS and 100 ERMS [60]. Not all the cores were evaluable, so in the end, 17 ARMS and 60 ERMS could be evaluated. Most of ARMS showed strong (Figure 9A, upper) or medium (Figure 9A, lower) L1CAM staining. In contrast, 95% of ERMS were negative. The H-score indicates how 85% of ARMS have high L1CAM expression, while the great majority of ERMS is negative. 

To investigate the relevance of *L1CAM* expression for clinical prognosis, we took advantage of an expression data set with survival information. We compared the overall survival of ARMS patients with mRNA levels of *L1CAM*. The best cut-off value was determined as 123.3 (range 3–390), and this revealed a significantly worse survival probability of ARMS patients with high *L1CAM* expression (*p* = 0.044; Figure 9C). Performing the same analysis on the whole cohort, including ARMS and ERMS patients, a cut-off of 57.3 resulted in a more significant logrank p-value of 0.0016, likely reflecting the better survival probability of ERMS vs. ARMS, and the *L1CAM* expression restricted to ARMS. In conclusion, L1CAM is highly expressed in the majority of ARMS, and within this histological subclass, higher expression of *L1CAM* seems to define a group of patients with even worse prognoses. Taken together, L1CAM targeted therapies could provide a therapeutic option for ARMS patients with very poor prognoses. 

## 3. Discussion

In this work, we identified 699 surface proteins by performing a surfaceome profiling by differential centrifugations enrichment of surface/membrane proteins and LC-MS detection with six FP-RMS cell lines, five FN-RMS cell lines, and three RMS PDXs. Ranking of the protein based on iTop3 expression analysis, mRNA expression, and expression in control normal MRC-5 fibroblasts and myoblasts, followed by statistical analysis and investigation of protein and mRNA expression in normal tissues, yielded nine surface proteins highly expressed in RMS and with low expression or absent in normal tissues: FGFR4, MEGF10, CD276, AGRL2, GPC4, JAM3, CADM2, NCAM1, and L1CAM. Expression of three of these candidates—AGRL2, JAM3, and L1CAM—on RMS cell lines was confirmed by FACS.

In this study, we found two well-known and investigated targets for RMS, FGFR4 [37,38,39] and N1CAM [22,23,24,25], validating our approach. CD276 (B7-H3) has also been recently shown to be consistently overexpressed in RMS with high expression in 92% of FP-RMS and with medium-high intensity in 100% of FN-RMS tumors [62]. CD276 expression is regulated by the fusion protein PAX3-FOXO1 found in FP-RMS [63], and the monoclonal antibody 8H9, binding to a wide spectrum of tumors, including RMS, was found to target CD276 [64,65]. The B7-H3-targeting antibody-drug conjugate m276-SL-PBD was potently effective against pediatric cancers in preclinical solid tumor models, including RMS [66]. Expression of CD276 on RMS cells was independently identified by another group by surfaceome profiling and was shown to be a mediator of immune evasion [51]. All these results confirm that CD276 is a relevant target for RMS.

**L1CAM.** Among the novel targets not previously associated with RMS before, targeting approaches are most advanced for L1CAM, which is highly and consistently overexpressed in neuroblastoma [67,68,69], ovarian cancer [70,71], and testicular germ cell tumors [72]. L1CAM was very early targeted with CAR T cells [73], and the effort to improve the CAR design continues (NCT02311621). Our results show that 85% of ARMS are strongly positive for L1CAM, and 95% of ERMS are negative. In a large study of 5155 tumors, expression of L1CAM was found in 50% alveolar (FP) RMS (n = 42) and in 15% embryonal (FN) RMS (n = 55) [74], confirming our observation. Here, we also show higher expression of L1CAM in ARMS compared to ERMS at the mRNA level. So far, no attention has been dedicated to targeting RMS with L1CAM antibodies or CAR T-cells, but our results would suggest that a small group of RMS patients with the poorest prognosis might benefit from such an approach.

**AGRL2**, or Adhesion G protein-coupled receptor L2, is an adhesion G-protein-coupled receptor (aGPCR) that was first described in 2000 [75], and whose function has not been well investigated yet. Like other aGPCRs, AGRL2 has been associated with cancer (reviewed in [76]). AGRL2 was found to be upregulated by transcriptome profiling in urothelial carcinoma [77]. To the best of our knowledge, its expression or function have never been investigated in RMS.

**CADM2**, cell adhesion molecule 2, belongs to the immunoglobulin superfamily and regulates cell adhesion, in particular synaptic assembly [78,79]. Its role in cancer is not completely clear: it is overexpressed in glioma [80], prostate cancer [81], and renal carcinoma [82], in which it can act as a tumor suppressor, but it promotes tumor metastasis in other cancers such as non-small cell lung cancer metastasis [83] and in hepatocellular carcinoma [84], with a role in epithelial to mesenchymal transition (EMT). In our analysis, CADM2 was significantly upregulated in all three RMS groups, FP-RMS, FN-RMS, and PDXs; and its expression in normal tissues was restricted to the brain. CADM2’s role and expression in RMS still need to be investigated.

**MEGF10** is a single transmembrane protein with particularly high expression in the CNS [85] and muscles [86,87]. In muscles, the expression seems to be restricted to satellite cells, the muscle progenitor cells, and MEGF10 mutations are associated with myopathies [88]. MEGF10 was among eleven RMS markers with high expression in RMS and low/no expression in normal peripheral blood or bone marrow to detect disseminated disease [89]. The overexpression of MEGF10 in RMS might be related to a block in myogenic differentiation [90]. Our analysis revealed a very restricted expression in normal tissues; however, CNS expression must be carefully evaluated to assess the safety of possible therapies targeting MEGF10. Overall, MEGF10 is a very appealing target for RMS therapy. 

**GPC4** belongs to the glypicans family, a family of heparan sulfate proteoglycans that are attached to the cell membrane via a glycosylphosphatidylinositol anchor, with a known role in cancer. So far, only GPC3 and GPC5 [91,92] have been associated with RMS (reviewed in [93]), but not GPC4. Several CAR constructs against glypicans have been developed, but so far, no GPC4 CAR has been reported [93], making the expression of GPC4 in RMS appealing for novel CAR design.

**JAM3**, or Junctional Adhesion Molecule (JAM) C, mediates heterotypic cell-cell interactions with its cognate receptor JAM2 [94,95]. JAM3 is involved in homing and mobilization of hematopoietic stem and progenitor cells within the bone marrow and by homology with zebrafish, might be involved in myocyte fusion [96,97]. JAMs are clearly involved in cell migration, polarization, and adhesion, and they are involved in cancer cells proliferation, migration, and invasion (reviewed in [98]). The function or expression of JAM3 in RMS has never been investigated.

Three additional promising targets could not be validated by FACS since we were not able to obtain a specific signal with the antibodies tested: EphA7 antibody clone 6C8G7 (Novus Biologicals, Centennial, CO; #NBP1-47425), FPRP clone 998107 (Novus Biologicals, #MAB100431), and SLC12A7 clone (R&D Systems, Minneapolis, MN; #MAB9030).

Among the RMS surface targets previously identified, HER2/ERBB2 is missing from our selected list. HER2 CAR T cells are being tested for RMS therapy, and one encouraging success has been reported [36]. HER2 was detected in FP-RMS cell lines and FN-RMS cell lines but not in PDXs; therefore, it was scored low and was also not selected in the following stringent analyses. A less stringent selection might have selected HER2/ERBB2, but also a higher number of proteins. Alternatively, the lack of identification of HER2/ERBB2 might reflect the heterogeneous expression of HER2 observed within tumors [99]. It is interesting to note, that HER3/ERBB3 was included in the Top100 list and showed significant expression in FP-RMS and FN-RMS cell lines, and PDXs. HER3/ERBB3 seems to be expressed in RMS more consistently than HER2/ERBB2 [99]. Although these results are dependent on the antibodies used and should be interpreted carefully, it is tempting to speculate that HER3 might be a good alternative to HER2/ERBB2 as a target for CAR T cell therapy in RMS.

One limitation of this type of study is posed by the availability of normal controls. Cultured primary cells like myoblasts, often used as a normal control for RMS, which express myogenic markers, or like fibroblasts, can be assumed to represent normal tissues; however, their surface expression can differ from normal tissues and can therefore serve only as a first screening tool. Proteomic databases representing ideally all human tissues are extremely useful to prioritize the targets with low expression in normal tissues. The challenge is how to compare our own data, e.g., surfaceome, with the reposited data that are derived from whole tissues and globally normalized. Detection of a protein in normal tissue does not disqualify it from being a viable therapeutic target. The difference in expression between tumor and normal tissue needs to be big enough to allow for selective targeting. Therefore, careful quantitative evaluation of the expression is mandatory. This is very important, since the identification of proteins exclusively expressed on tumors is a very rare event. The final evaluation of the therapeutic window needs to be performed in more complex model systems, non-human primates, and eventually in patients.

In conclusion, surfaceome profiling of cultured tumor cells is a very powerful tool to identify novel putative cell surface targets for antibody-based therapies, such as CAR T-cell therapy. Here, we confirm FGFR4, NCAM1, and CD276 as specific RMS targets, and identify AGRL2, JAM3, MEGF10, as promising candidates. In particular, high L1CAM expression observed in the aggressive ARMS histological subtype, and its inverse correlation with survival, support further investigation of L1CAM targeted therapies for patients with dismal prognosis.

## 4. Materials and Methods

### 4.1. Cell Culture

Human RMS cell lines RD, Rh4, Rh5, Rh18, Rh28, Rh30, Rh36, JR, RMS, RUCH-3, and TTC-442 were kindly provided by Prof. Beat Schäfer, University Children’s Hospital Zurich, Switzerland. PDX IC-pPDX-104 (referred to as PDX_104), IC-pPDX-29 (referred to as PDX_29), and IC-pPDX-35 (referred to as PDX_35) were established at the Institut Curie in Paris, France as described in [100,101]. IC-pPDX-29: 14-year-old female with recurrent primary paravertebral ARMS (*PAX3-FOXO1* translocation status unknown). IC-pPDX-35: 13-year-old male with recurrent metastatic ARMS with *PAX3-FOXO1* translocation to the mediastinum. IC-pPDX-104: 7-year-old female with recurrent primary ARMS with *PAX3-FOXO1* translocation to the tibia. Immortalized human healthy primary myoblasts KM155C25Dist (referred to as myoblasts), kindly provided by the platform for immortalization of human cells from the Institut de Myologie (Sorbonne University, Paris, France), and the MRC-5 cell line (ATCC) were used as negative controls. All cell lines, with the exception of human myoblasts, were cultured in DMEM (BioConcept, Allschwil, Switzerland; #1-26F01-I), supplemented with 10% fetal bovine serum (FBS, Thermo Fisher Scientific—Gibco, Zug, Switzerland; #10270106), L-glutamine 2 mM (BioConcept, #5-10K00-H) and 100 U/mL Penicillin-Streptomycin (BioConcept, #4-01F00-H) at 37 °C and 5% CO_2_ in a humidified incubator. Myoblasts were cultured in Skeletal Muscle Cell Growth Medium (PromoCell, Heidelberg, Germany; #C-23060) supplemented with Skeletal Muscle Cell Growth Medium SupplementMix (PromoCell, #C-39365). IC-pPDX-104, IC-pPDX-29, and IC-pPDX-35 were cultured in five P15 dishes, precoated with 1:10 diluted matrigel (Corning, Amsterdam, The Netherlands; #354234) into 3 mL of precooled neurobasal medium (Gibco, #10888022), supplemented with Glutamax (Thermo Fisher Scientific—Gibco, #35050), 100 U/mL Penicillin-Streptomycin (BioConcept, #4-01F00-H), 2x B-27 (Thermo Fisher Scientific—Life Technologies, #17504044), 20 ng/mL bFGF (PeproTech, #AF-100-18B), 20 ng/mL EGF (PeproTech, London, UK; #AF-100-15). 

### 4.2. Cell Surface Proteins Isolation

Membrane/surface proteins were enriched with two methods: (1) Cell surface biotinylation and isolation (Thermo Fisher Scientific, #A44390), following the manufacturer’s instructions; (2) with a two-step protocol of ultracentrifugation and high salt washes [52]. Briefly, 1 × 10^7^ cells were seeded on five P15 dishes. On the day of the experiment, 80–90% confluent cells were gently washed twice with PBS at RT, collected with a scraper, and centrifuged at 700× *g* at RT for 5 min. After resuspension in 1 mL cold hypotonic buffer (50 mM Mannitol, 5 mM HEPES, pH 7.4), the cells were homogenized with 1 min sonication (10% duty cycle, Branson Sonifer 250, Thermo Fisher Scientific) and centrifuged at 600× *g* at 4 °C for 10 min. The supernatant was then processed following differential centrifugations: 15,000× *g*, 4 °C for 5 min; wash in 10 mM CaCl_2_; shaking at 4 °C for 10 min; 3000× *g* at RT for 15 min; 48,000× *g* for 30 min at RT; wash in 1 M KCl; 48,000× *g* at RT for 30 min; wash in 0.5 mL 100 mM Na_2_CO_3_; 48,000× *g* at RT for 30 min. Next, all the samples were resuspended in 20 µL Laemmli buffer (62.5 mM TrisHCl, pH 6.8, 1% SDS, 10% Glycerol, 40 mM DTT) and separated by 1D gel-electrophoresis, 1.5 cm long gel-migration. For all the cell lines, three replicates were obtained. The SDS gel was fixed with 10% glacial acetic acid/40% EtOH, stained with 0.1% Brilliant Blue G in 45% EtOH/10% acetic acid and destained with 10% glacial acetic acid/40% EtOH in order to visualize the protein bands. Each lane was cut in four horizontal bands, and each band was further cut into six gel cubes. The six pieces of gel were then stored in 20% EtOH at 4 °C until processing.

### 4.3. In-Gel Digestion and Mass Spectrometry (MS)

MS experiments were performed in collaboration with the DBMR proteomics core facility (University of Bern). Proteins were in-gel digested as previously described [102]. Digests were loaded onto a precolumn (C18 PepMap 100, 5 µm, 100 A, 300 µm i.d. × 5 mm length, Thermo Fisher Scientific) at a flow rate of 50 µL/min with solvent C (0.05% TFA in water/acetonitrile 98:2). After loading, peptides were eluted in back flush mode onto a homemade C18 CSH Waters column (1.7 μm, 130 Å, 75 μm × 20 cm) by applying a 40 min gradient of 5% acetonitrile to 40% in water, 0.1% formic acid, at a flow rate of 250 nL/min. The column effluent was directly coupled to a Fusion LUMOS mass spectrometer (Thermo Fischer Scientific) via a nano-spray ESI source. Data acquisition was made in data-dependent mode with precursor ion scans recorded in the orbitrap with a resolution of 120,000 (at *m*/*z* = 250) parallel to top speed fragment spectra of the most intense precursor ions in the Linear trap for a cycle time of 3 s maximum. 

The samples were searched and quantified with MaxQuant [103] version 2.0.1.0, using the SWISS-PROT [104] Homo sapiens database (April 2021 release) containing isoforms, and to which common contaminants were added. Search parameters were the following: enzyme was set to strict trypsin, with a maximum of three missed cleavages allowed; the first search peptide tolerance was set to 10 ppm, and the MS/MS match tolerance to 0.4 Da; carbamidomethylation on cysteine was set as a fixed modification, while methionine oxidation, asparagine, and glutamine deamidation, and protein N-terminal acetylation were set as variable modifications. The matches between runs were enabled, with the corresponding fractions labeled 1 to 4. The Top3 values were calculated by first normalizing peptide forms with variance stabilization normalization [105] and imputing them (see below) before summing the top three intensities. 

Imputation at the peptide level was performed as follows: if there was at most one non-zero value in a group of replicates, then the remaining missing values were drawn from a Gaussian distribution of width 0.3 times the sample standard deviation and centered at the sample distribution mean minus 2.8 times the sample standard deviation; any remaining missing values were imputed by the Maximum Likelihood Estimation (MLE) method [106].

### 4.4. MS Data Processing and Data Mining

MS-derived data were inspected with the Panther database (www.pantherdb.org (accessed on 15 September 2022)) to evaluate the amount of membrane-associated proteins and validate the experiments. To select membrane/surface proteins with higher confidence, two published lists of predicted/annotated membrane/surface proteins were used. List A, a list of 2886 predicted and experimentally validated surface proteins by SURFY with an accuracy of 93.5%, which is included in the Cell Surface Protein Atlas (CSPA), published by Bausch et al. (Supplementary Information, List A) [53]. List B [107], a comprehensive list of 7643 membrane/surface proteins generated bioinformatically, by pooling annotated surface proteins from Gene Ontology [108], transmembrane proteins predicted by hidden Markov models (TMHMM) [109], and glycosylphosphatidylinositol (GPI)-anchored proteins [107] (Supplementary Information, List B).

Subcellular localization of the putative targets was verified by using Genecards source (www.genecards.org (accessed on 15 September 2022)); protein expression in normal tissues was evaluated with Human Protein Atlas database (www.proteinatlas.org (accessed on 15 September 2022)) checking RNA expression (nTPM) and protein expression (score). The UniProt Knowledgebase was used to confirm single candidates as membrane proteins (www.uniprot.org (accessed on 15 September 2022)). Briefly, membrane/surface proteins classified in UniProt as “reviewed” were sorted by the keywords “Homo sapiens” in Taxonomy and “Transmembrane” in Subcellular location searching fields, and the corresponding gene names were converted into UniProt KB ID.

### 4.5. Scoring Strategy for Sorted Membrane/Surface Proteins

The membrane/surface proteins extracted from the MS data were further processed to determine the Top100 upregulated surface proteins. A stringent scoring was designed to assign lower grades to the most RMS-specific candidates, expressed at the highest levels (Table 1).

### 4.6. Statistical Analysis

Differential expression by moderated t-statistics and significance evaluation was performed following Uldry et al., 2022 [110], with a minimum of log_2_ fold change of 1 and a maximum adjusted *p*-value of 0.05 for each individual comparison using the imputed Top3 intensities for each set of cells, Results for C-list proteins were summarized by plotting on the x-axis the average log_2_ fold changes between each cell of the set and MRC-5 and Myoblasts, and on the y-axis, the average of the corresponding moderated t-statistics of the comparisons. Proteins for which the moderated t-statistics were above 2.132 (95th percentile of the corresponding Student’s distribution) in all three sets of cells were considered of interest. Graphs were generated with R. The linear mixed model (LMM) was derived from the R implementation DREAM [56] and was used to perform a statistical evaluation of all the respective FP-RMS, FN-RMS, and PDXs groups versus the controls while accounting for the fact that each subgroup of replicates are repeated measurements. Differential expression and significance evaluation were performed as above. Volcano plots were generated with the online tool VolcanoNoseR [111].

### 4.7. Transcriptomics Data Analysis

The mRNA expression data of the genes corresponding to the Top100 putative targets for RMS tumors and normal tissues were obtained from the RMS whole-transcriptome sequencing data set (dbGaP Study Accession: phs000720.v3.p1), reported in 2021 by Brohl et al. [49].

### 4.8. Scoring Strategy for mRNA Data from RMS Tumors

The mRNA levels of the genes for the Top100 proteins were ranked by applying the scores in Table 2.

### 4.9. Antibodies

Primary antibodies for Flow Cytometry: mouse anti-latrophilin 2/AGRL2/LPHN2 (1 µg/10^6^ cells; R&D Systems, #MAB105881-SP); mouse anti-glypican 4 (1 µg/10^6^ cells; R&D Systems, MAB9195-SP); mouse anti-JAM3 (1 µg/10^6^ cells; R&D SYSTEMS, MAB11891-SP); PE-conjugated anti-L1CAM, (1:20; Miltenyi Biotec, Bergisch Gladbach, Germany; #130-100-691); PE-conjugated anti-FGFR4 (1:100; Biolegend, London, UK; #324306). Secondary antibodies: FITC Goat anti-mouse IgG antibody (1:500; BioLegend, #405305). Isotype controls: mouse FITC-conjugated IgG1, κ Isotype Ctrl Antibody (1:100; BioLegend, #400108); rabbit PE-conjugated (1:100; R&D Systems, #AB-105-C).

### 4.10. Flow Cytometry Analysis

Cells were detached with Accutase (Thermo Fisher Scientific) for 10 min at 37 °C, washed with PBS, and counted. 100,000 cells were incubated in 100 µL FACS buffer (2% BSA in PBS) with the primary antibodies at the optimized concentrations for 30 min at RT. Flow cytometry measurements were performed with a CytoFLEX device (Beckman Coulter, Krefeld, Germany). The results were analyzed by FlowJo v10.8.1 Software (BD Life Sciences, Allschwil, Switzerland).

### 4.11. Tissue Microarrays

A tissue microarray with 248 cores from 124 RMS tumors (24 ARMS, of which 17 with known FOXO1 gene rearrangements and 100 ERMS) was constructed [60]. Tumors used were collected at the University Hospital Zurich, Switzerland and at the Kiel Pediatric Tumor Registry, Kiel, Germany. Immunohistochemistry was performed essentially as described in [72] by using the monoclonal antibody anti-L1CAM (clone 14.10, directed to the ectodomain, 1:200).

### 4.12. Survival Analysis

The correlation between L1CAM mRNA expression levels and RMS survival was analyzed with the dataset “Rhabdomyosarcoma Davicioni 147” publicly available through the R2 Genomics Analysis and Visualization Platform (http://r2.amc.nl; ps_avgpres_rmstriche147_u133a (accessed on 12 January 2023)), derived from a comprehensive analysis of 147 RMS samples [39], and survival data were obtained from the supplementary Tables in Davicioni et al. [112]. The Kaplan–Meier plot was generated with https://kmplot.com (accessed on 15 Juanuary 2023) autoselecting for best cut-off and performing univariate Cox regression as described [113]. Significance was computed using the Cox–Mantel (logrank) test [61].

## Figures and Tables

**Table 1:** Scoring for membrane/surface proteins extracted from the proteomics data analysis.

Score for Number of Cell Lines		Score for iTop3 Mean in All the Cell Lines	
Number of Cell Lines	Score	iTop3 Range	Score
14	0	>2,500,000,000	0
13	0.5	2,500,000,000–1,000,000,000	1
12	1.5	1,000,000,000–500,000,000	2
11	3	500,000,000–250,000,000	3
10	5	250,000,000–100,000,000	4
9	7.5	100,000,000–50,000,000	5
8	10	50,000,000–25,000,000	6
7	15	25,000,000–10,000,000	7
≤6	30	10,000,000–5,000,000	8
Score for Detection in the Controls		Score for Log_2_(FoldChange)	
In 0/2 Ctrl cell lines	0	If Log_2_(FC) = 10	0
In 1/2 Ctrl cell lines	2.5	If 6 ≤ Log_2_(FC) < 10	0.5
In 2/2 Ctrl cell lines	5	If 5 ≤ Log_2_(FC) < 6	1
Bonus for Expression in PDXs		If 4 ≤ Log_2_(FC) < 5	1.5
In 3/3 PDXs	0	If 3 ≤ Log_2_(FC) < 4	2
In 2/3 PDXs	0.5	If 2 ≤ Log_2_(FC) < 3	2.5
In 1/3 PDXs	1	If 1 ≤ Log_2_(FC) < 2	3
In No PDXs	1.5	If Log_2_(FC) < 1	3.5
Bonus for RMS Specific Expression and High Abundance in All the Cell Lines			
If Mean iTop3 expression > 10^7^		−3	
Log_2_(FC) = 10 *			
CL ≥ 13			

* The Log_2_(FC) of proteins not expressed in the controls received a maximum fixed score of 10 for the scoring strategy. FC: Fold Change; CL: number of Cell Lines.

**Table 2:** Scoring strategy for mRNA expression.

Score for Expression in Normal Tissues		Score for Expression in RMS Tumors	
FPKM	Score	FPKM	Score
<5	0.0	>500	0.0
5–10	0.5	250–500	0.5
10–50	1.5	100–250	1.5
50–100	3.0	50–100	3.0
100–250	5.0	25–50	5.0
250–500	7.5	10–25	7.5
>500	10.0	5–10	10.0
		<5	15.0
Score for Expression in the Controls *		Score for Log_2_(FC)	
In 0 Ctrls	0.00	If Log_2_(FC) ≥ 5	0
In 1 Ctrls	0.25	If 4 ≤ Log_2_(FC) < 5	1
In 2 Ctrls	0.50	If 3 ≤ Log_2_(FC) < 4	2
In 3 Ctrls	0.75	If 2 ≤ Log_2_(FC) < 3	3
In 4 Ctrls	1.00	If 1.5 ≤ Log_2_(FC) < 2	4
In 5 Ctrls	1.25	If 1 ≤ Log_2_(FC) < 1.5	5
		If Log_2_(FC) < 1	10

* Controls tissues: brain, heart, kidney, liver, and lung.

## Data Availability

Proteomics data have been deposited to proteomeXchange.org (identifier PXD039480) [114].

## Supplementary Materials

The following supporting information can be downloaded at: https://www.mdpi.com/article/10.3390/ijms24032601/s1.

## Appendix A

**Table A1:** List C: “Top100” list of surface proteins ranked by the criteria of the MS scoring strategy.

#	UniProt ID	Entry Name	Protein Name (Alternative Names)	Mean iTop3 in RMS	Mean iTop3 in Ctrls	Log2 (Ratio RMS/Ctrls)	Score
1	O95490-2	ADGRL2	Isoform 2 of Adhesion G protein-coupled receptor L2 OS = Homo sapiens OX = 9606 GN = ADGRL2; Isoform 5 of Adhesion G protein-coupled receptor L2 OS = Homo sapiens OX = 9606 GN = ADGRL2	1.10 × 10^8^	0.00	10.000	1
2	O75487	GPC4	Glypican-4 OS = Homo sapiens OX = 9606 GN = GPC4 PE = 1 SV = 4; Isoform 2 of Glypican-4 OS = Homo sapiens OX = 9606 GN = GPC4	4.72 × 10^7^	0.00	10.000	3
3	Q15375-2	EPHA7	Isoform 2 of Ephrin type-A receptor 7 OS = Homo sapiens OX = 9606 GN = EPHA7; Isoform 4 of Ephrin type-A receptor 7 OS = Homo sapiens OX = 9606 GN = EPHA7; Ephrin type-A receptor 7	2.97 × 10^7^	0.00	10.000	3
4	P32004-3	L1CAM	Isoform 3 of Neural cell adhesion molecule L1 OS = Homo sapiens OX = 9606 GN = L1CAM	8.29 × 10^7^	0.00	10.000	4
5	Q8TEM1	NUP210	Nuclear pore membrane glycoprotein 210 OS = Homo sapiens OX = 9606 GN = NUP210 PE = 1 SV = 3	7.10 × 10^7^	3.47 × 10^5^	7.677	5
6	Q9P2B2	PTGFRN	Prostaglandin F2 receptor negative regulator OS = Homo sapiens OX = 9606 GN = PTGFRN PE = 1 SV = 2	1.35 × 10^8^	2.18 × 10^6^	5.958	5
7	P13591	NCAM1	Neural cell adhesion molecule 1 OS = Homo sapiens OX = 9606 GN = NCAM1 PE = 1 SV = 3	7.02 × 10^8^	0.00	10.000	6
8	Q15223	NECTIN1	Nectin-1 OS = Homo sapiens OX = 9606 GN = NECTIN1 PE = 1 SV = 3	8.31 × 10^7^	0.00	10.000	8.5
9	Q8N3J6-2	CADM2	Isoform 2 of Cell adhesion molecule 2 OS = Homo sapiens OX = 9606 GN = CADM2; Isoform 3 of Cell adhesion molecule 2 OS = Homo sapiens OX = 9606 GN = CADM2; Cell adhesion molecule 2	5.24 × 10^7^	0.00	10.000	10
10	P22455	FGFR4	Fibroblast growth factor receptor 4 OS = Homo sapiens OX = 9606 GN = FGFR4 PE = 1 SV = 2; Isoform 2 of Fibroblast growth factor receptor 4 OS = Homo sapiens OX = 9606 GN = FGFR4	3.02 × 10^8^	8.76 × 10^6^	5.105	10.5
11	P52803	EFNA5	Ephrin-A5 OS = Homo sapiens OX = 9606 GN = EFNA5 PE = 1 SV = 1	1.03 × 10^7^	0.00	10.000	10.5
12	P54826	GAS1	Growth arrest-specific protein 1 OS = Homo sapiens OX = 9606 GN = GAS1 PE = 2 SV = 2	2.27 × 10^7^	0.00	10.000	10.5
13	P78310	CXADR	Coxsackievirus and adenovirus receptor OS = Homo sapiens OX = 9606 GN = CXADR PE = 1 SV = 1; Isoform 7 of Coxsackievirus and adenovirus receptor OS = Homo sapiens OX = 9606 GN = CXADR	4.98 × 10^7^	0.00	10.000	10.5
14	Q9Y5Y0	FLVCR1	Feline leukemia virus subgroup C receptor-related protein 1 OS = Homo sapiens OX = 9606 GN = FLVCR1 PE = 1 SV = 1	6.64 × 10^7^	2.07 × 10^6^	5.002	10.5
15	Q9BSA4	TTYH2	Protein tweety homolog 2 OS = Homo sapiens OX = 9606 GN = TTYH2 PE = 1 SV = 3	4.05 × 10^7^	0.00	10.000	11
16	Q9Y666	SLC12A7	Solute carrier family 12 member 7 OS = Homo sapiens OX = 9606 GN = SLC12A7 PE = 1 SV = 3	7.23 × 10^7^	5.88 × 10^6^	3.618	11
17	Q6ZRP7	QSOX2	Sulfhydryl oxidase 2 OS = Homo sapiens OX = 9606 GN = QSOX2 PE = 1 SV = 3	1.76 × 10^8^	2.66 × 10^7^	2.724	12
18	Q9P0T7	TMEM9	Proton-transporting V-type ATPase complex assembly regulator TMEM9 OS = Homo sapiens OX = 9606 GN = TMEM9 PE = 1 SV = 1	1.54 × 10^8^	2.44 × 10^7^	2.662	12
19	Q9Y289	SLC5A6	Sodium-dependent multivitamin transporter OS = Homo sapiens OX = 9606 GN = SLC5A6 PE = 2 SV = 2	5.08 × 10^7^	2.95 × 10^6^	4.105	12
20	O43155	FLRT2	Leucine-rich repeat transmembrane protein FLRT2 OS = Homo sapiens OX = 9606 GN = FLRT2 PE = 1 SV = 1	2.07 × 10^7^	0.00	10.000	12.5
21	O75051	PLXNA2	Plexin-A2 OS = Homo sapiens OX = 9606 GN = PLXNA2 PE = 1 SV = 4	5.39 × 10^7^	1.03 × 10^7^	2.382	13
22	P16144-4	ITGB4	Isoform Beta-4D of Integrin beta-4 OS = Homo sapiens OX = 9606 GN = ITGB4; Isoform Beta-4A of Integrin beta-4 OS = Homo sapiens OX = 9606 GN = ITGB4	5.63 × 10^7^	0.00	10.000	13
23	P50993	ATP1A2	Sodium/potassium-transporting ATPase subunit alpha-2 OS = Homo sapiens OX = 9606 GN = ATP1A2 PE = 1 SV = 1	1.16 × 10^7^	0.00	10.000	13
24	Q6N075	MFSD5	Molybdate-anion transporter OS = Homo sapiens OX = 9606 GN = MFSD5 PE = 1 SV = 2; Isoform 2 of Molybdate-anion transporter OS = Homo sapiens OX = 9606 GN = MFSD5	9.47 × 10^6^	0.00	10.000	13
25	Q8NFZ8	CADM4	Cell adhesion molecule 4 OS = Homo sapiens OX = 9606 GN = CADM4 PE = 1 SV = 1	1.05 × 10^7^	0.00	10.000	13
26	Q99808	SLC29A1	Equilibrative nucleoside transporter 1 OS = Homo sapiens OX = 9606 GN = SLC29A1 PE = 1 SV = 3	3.24 × 10^8^	6.67 × 10^7^	2.281	13
27	Q9BX67	JAM3	Junctional adhesion molecule C OS = Homo sapiens OX = 9606 GN = JAM3 PE = 1 SV = 1; Isoform 2 of Junctional adhesion molecule C OS = Homo sapiens OX = 9606 GN = JAM3	2.60 × 10^8^	5.58 × 10^7^	2.220	13
28	P13637	ATP1A3	Sodium/potassium-transporting ATPase subunit alpha-3 OS = Homo sapiens OX = 9606 GN = ATP1A3 PE = 1 SV = 3	2.87 × 10^7^	0.00	10.000	13.5
29	P49810-2	PSEN2	Isoform 2 of Presenilin-2 OS = Homo sapiens OX = 9606 GN = PSEN2; Isoform 3 of Presenilin-2 OS = Homo sapiens OX = 9606 GN = PSEN2	3.57 × 10^7^	9.20 × 10^5^	5.277	13.5
30	Q9H2E6	SEMA6A	Semaphorin-6A OS = Homo sapiens OX = 9606 GN = SEMA6A PE = 1 SV = 2	1.40 × 10^7^	0.00	10.000	13.5
31	O60245	PCDH7	Protocadherin-7 OS = Homo sapiens OX = 9606 GN = PCDH7 PE = 1 SV = 2	1.87 × 10^7^	8.32 × 10^5^	4.492	14
32	O95858	TSPAN15	Tetraspanin-15 OS = Homo sapiens OX = 9606 GN = TSPAN15 PE = 1 SV = 1	9.18 × 10^6^	0.00	10.000	14
33	P20645	M6PR	Cation-dependent mannose-6-phosphate receptor OS = Homo sapiens OX = 9606 GN = M6PR PE = 1 SV = 1	1.08 × 10^9^	8.52 × 10^8^	0.347	14
34	Q13491-4	GPM6B	Isoform 4 of Neuronal membrane glycoprotein M6-b OS = Homo sapiens OX = 9606 GN = GPM6B	3.04 × 10^7^	0.00	10.000	14
35	Q14542	SLC29A2	Equilibrative nucleoside transporter 2 OS = Homo sapiens OX = 9606 GN = SLC29A2 PE = 1 SV = 3	1.95 × 10^7^	0.00	10.000	14
36	Q15043-2	SLC39A14	Isoform 3 of Metal cation symporter ZIP14 OS = Homo sapiens OX = 9606 GN = SLC39A14	2.31 × 10^8^	5.48 × 10^7^	2.079	14
37	Q92823-3	NRCAM	Isoform 3 of Neuronal cell adhesion molecule OS = Homo sapiens OX = 9606 GN = NRCAM	3.76 × 10^7^	0.00	10.000	14
38	Q96KG7	MEGF10	Multiple epidermal growth factor-like domains protein 10 OS = Homo sapiens OX = 9606 GN = MEGF10 PE = 1 SV = 1	1.77 × 10^7^	4.02 × 10^6^	2.142	14
39	Q9H0V9	LMAN2L	VIP36-like protein OS = Homo sapiens OX = 9606 GN = LMAN2L PE = 1 SV = 1; Isoform 2 of VIP36-like protein OS = Homo sapiens OX = 9606 GN = LMAN2L	7.37 × 10^7^	2.20 × 10^7^	1.747	14
40	P29972	AQP1	Aquaporin-1 OS = Homo sapiens OX = 9606 GN = AQP1 PE = 1 SV = 3	1.86 × 10^7^	0.00	10.000	15
41	P53985	SLC16A1	Monocarboxylate transporter 1 OS = Homo sapiens OX = 9606 GN = SLC16A1 PE = 1 SV = 3	4.93 × 10^8^	1.94 × 10^8^	1.346	15
42	Q12907	LMAN2	Vesicular integral-membrane protein VIP36 OS = Homo sapiens OX = 9606 GN = LMAN2 PE = 1 SV = 1	6.67 × 10^8^	6.11 × 10^8^	0.128	15
43	Q5T3U5-2	ABCC10	Isoform 2 of ATP-binding cassette sub-family C member 10 OS = Homo sapiens OX = 9606 GN = ABCC10; ATP-binding cassette sub-family C member 10 OS = Homo sapiens OX = 9606 GN = ABCC10 PE = 1 SV = 1	1.13 × 10^7^	0.00	10.000	15
44	Q7Z3C6	ATG9A	Autophagy-related protein 9A OS = Homo sapiens OX = 9606 GN = ATG9A PE = 1 SV = 3	7.88 × 10^7^	1.69 × 10^7^	2.222	15
45	Q92544	TM9SF4	Transmembrane 9 superfamily member 4 OS = Homo sapiens OX = 9606 GN = TM9SF4 PE = 1 SV = 2	7.35 × 10^8^	3.90 × 10^8^	0.913	15
46	O94856-4	NFASC	Isoform 4 of Neurofascin OS = Homo sapiens OX = 9606 GN = NFASC	5.07 × 10^7^	0.00	10.000	15.5
47	P0C7U0	ELFN1	Protein ELFN1 OS = Homo sapiens OX = 9606 GN = ELFN1 PE = 1 SV = 2	5.88 × 10^7^	0.00	10.000	15.5
48	P32418-2	SLC8A1	Isoform 3 of Sodium/calcium exchanger 1 OS = Homo sapiens OX = 9606 GN = SLC8A1	4.50 × 10^9^	3.46 × 10^7^	7.022	15.5
49	P04843	RPN1	Dolichyl-diphosphooligosaccharide—protein glycosyltransferase subunit 1 OS = Homo sapiens OX = 9606 GN = RPN1 PE = 1 SV = 1	1.43 × 10^9^	3.21 × 10^9^	−1.166	16
50	P05023-3	ATP1A1	Isoform 3 of Sodium/potassium-transporting ATPase subunit alpha-1 OS = Homo sapiens OX = 9606 GN = ATP1A1	1.51 × 10^9^	1.81 × 10^9^	−0.257	16
51	P05556	ITGB1	Integrin beta-1 OS = Homo sapiens OX = 9606 GN = ITGB1 PE = 1 SV = 2	1.69 × 10^9^	5.95 × 10^9^	−1.818	16
52	P35613	BSG	Basigin OS = Homo sapiens OX = 9606 GN = BSG PE = 1 SV = 2	1.17 × 10^9^	7.28 × 10^8^	0.682	16
53	P54709	ATP1B3	Sodium/potassium-transporting ATPase subunit beta-3 OS = Homo sapiens OX = 9606 GN = ATP1B3 PE = 1 SV = 1	1.19 × 10^9^	7.59 × 10^8^	0.649	16
54	P56746	CLDN15	Claudin-15 OS = Homo sapiens OX = 9606 GN = CLDN15 PE = 1 SV = 1	4.67 × 10^6^	0.00	10.000	16
55	Q99805	TM9SF2	Transmembrane 9 superfamily member 2 OS = Homo sapiens OX = 9606 GN = TM9SF2 PE = 1 SV = 1	3.08 × 10^8^	2.88 × 10^8^	0.094	16
56	Q9BZM6	ULBP1	UL16-binding protein 1 OS = Homo sapiens OX = 9606 GN = ULBP1 PE = 1 SV = 1	5.59 × 10^6^	0.00	10.000	16
57	Q9H8M5	CNNM2	Metal transporter CNNM2 OS = Homo sapiens OX = 9606 GN = CNNM2 PE = 1 SV = 2	4.54 × 10^6^	0.00	10.000	16
58	Q9P273	TENM3	Teneurin-3 OS = Homo sapiens OX = 9606 GN = TENM3 PE = 2 SV = 3	1.90 × 10^7^	5.83 × 10^6^	1.705	16
59	O15431	SLC31A1	High affinity copper uptake protein 1 OS = Homo sapiens OX = 9606 GN = SLC31A1 PE = 1 SV = 1	1.31 × 10^8^	1.21 × 10^8^	0.117	17
60	P11117	ACP2	Lysosomal acid phosphatase OS = Homo sapiens OX = 9606 GN = ACP2 PE = 1 SV = 3	1.26 × 10^8^	1.14 × 10^8^	0.140	17
61	P19256-2	CD58	Isoform 2 of Lymphocyte function-associated antigen 3 OS = Homo sapiens OX = 9606 GN = CD58	2.86 × 10^6^	0.00	10.000	17
62	P41143	OPRD1	Delta-type opioid receptor OS = Homo sapiens OX = 9606 GN = OPRD1 PE = 1 SV = 4	1.17 × 10^7^	0.00	10.000	17
63	P51654	GPC3	Glypican-3 OS = Homo sapiens OX = 9606 GN = GPC3 PE = 1 SV = 1	3.15 × 10^7^	0.00	10.000	17
64	Q13308	PTK7	Inactive tyrosine-protein kinase 7 OS = Homo sapiens OX = 9606 GN = PTK7 PE = 1 SV = 2	2.36 × 10^8^	2.06 × 10^8^	0.193	17
65	Q14108	SCARB2	Lysosome membrane protein 2 OS = Homo sapiens OX = 9606 GN = SCARB2 PE = 1 SV = 2	8.23 × 10^8^	1.60 × 10^9^	−0.962	17
66	Q15758	SLC1A5	Neutral amino acid transporter B(0) OS = Homo sapiens OX = 9606 GN = SLC1A5 PE = 1 SV = 2	5.35 × 10^8^	4.48 × 10^8^	0.254	17
67	Q5ZPR3	CD276	CD276 antigen OS = Homo sapiens OX = 9606 GN = CD276 PE = 1 SV = 1	1.83 × 10^8^	1.34 × 10^8^	0.449	17
68	Q68DH5	LMBRD2	G-protein coupled receptor-associated protein LMBRD2 OS = Homo sapiens OX = 9606 GN = LMBRD2 PE = 1 SV = 1	4.27 × 10^7^	1.40 × 10^7^	1.609	17
69	Q8NFM7-4	IL17RD	Isoform 4 of Interleukin-17 receptor D OS = Homo sapiens OX = 9606 GN = IL17RD	3.41 × 10^7^	0.00	10.000	17
70	Q8WY21-3	SORCS1	Isoform 3 of VPS10 domain-containing receptor SorCS1 OS = Homo sapiens OX = 9606 GN = SORCS1;	3.79 × 10^7^	4.25 × 10^6^	3.160	17
71	Q92542	NCSTN	Nicastrin OS = Homo sapiens OX = 9606 GN = NCSTN PE = 1 SV = 2; Isoform 2 of Nicastrin OS = Homo sapiens OX = 9606 GN = NCSTN	5.52 × 10^8^	4.31 × 10^8^	0.358	17
72	Q9Y625	GPC6	Glypican-6 OS = Homo sapiens OX = 9606 GN = GPC6 PE = 1 SV = 1	6.19 × 10^7^	9.59 × 10^6^	2.691	17
73	O15031	PLXNB2	Plexin-B2 OS = Homo sapiens OX = 9606 GN = PLXNB2 PE = 1 SV = 3	2.73 × 10^8^	5.04 × 10^8^	−0.886	18
74	O15321-2	TM9SF1	Isoform 2 of Transmembrane 9 superfamily member 1 OS = Homo sapiens OX = 9606 GN = TM9SF1; Transmembrane 9 superfamily member 1 OS = Homo sapiens OX = 9606 GN = TM9SF1 PE = 2 SV = 2	7.34 × 10^7^	5.57 × 10^7^	0.397	18
75	O75954	TSPAN9	Tetraspanin-9 OS = Homo sapiens OX = 9606 GN = TSPAN9 PE = 1 SV = 1	6.23 × 10^7^	4.35 × 10^7^	0.518	18
76	P05026-2	ATP1B1	Isoform 2 of Sodium/potassium-transporting ATPase subunit beta-1 OS = Homo sapiens OX = 9606 GN = ATP1B1; Sodium/potassium-transporting ATPase subunit beta-1 OS = Homo sapiens OX = 9606 GN = ATP1B1 PE = 1 SV = 1	2.61 × 10^8^	4.10 × 10^8^	−0.652	18
77	P11166	SLC2A1	Solute carrier family 2, facilitated glucose transporter member 1 OS = Homo sapiens OX = 9606 GN = SLC2A1 PE = 1 SV = 2	3.30 × 10^8^	3.01 × 10^9^	−3.189	18
78	P21860-4	ERBB3	Isoform 4 of Receptor tyrosine-protein kinase erbB-3 OS = Homo sapiens OX = 9606 GN = ERBB3; Receptor tyrosine-protein kinase erbB-3 OS = Homo sapiens OX = 9606 GN = ERBB3 PE = 1 SV = 1	2.72 × 10^7^	5.26 × 10^5^	5.694	18
79	P22897-2	MRC1	Isoform 2 of Macrophage mannose receptor 1 OS = Homo sapiens OX = 9606 GN = MRC1; Macrophage mannose receptor 1 OS = Homo sapiens OX = 9606 GN = MRC1 PE = 1 SV = 1	1.22 × 10^7^	0.00	10.000	18
80	P23229-4	ITGA6	Isoform Alpha-6X2A of Integrin alpha-6 OS = Homo sapiens OX = 9606 GN = ITGA6	3.29 × 10^8^	3.60 × 10^8^	−0.130	18
81	P29317	EPHA2	Ephrin type-A receptor 2 OS = Homo sapiens OX = 9606 GN = EPHA2 PE = 1 SV = 2	3.58 × 10^8^	6.30 × 10^8^	−0.817	18
82	P30825	SLC7A1	High affinity cationic amino acid transporter 1 OS = Homo sapiens OX = 9606 GN = SLC7A1 PE = 1 SV = 1	1.97 × 10^8^	7.64 × 10^7^	1.363	18
83	P54753	EPHB3	Ephrin type-B receptor 3 OS = Homo sapiens OX = 9606 GN = EPHB3 PE = 1 SV = 2	5.16 × 10^7^	5.00 × 10^7^	0.044	18
84	Q8IWA5	SLC44A2	Choline transporter-like protein 2 OS = Homo sapiens OX = 9606 GN = SLC44A2 PE = 1 SV = 3;	2.57 × 10^8^	2.33 × 10^8^	0.138	18
85	Q8NE01	CNNM3	Metal transporter CNNM3 OS = Homo sapiens OX = 9606 GN = CNNM3 PE = 1 SV = 1	2.31 × 10^7^	5.81 × 10^6^	1.991	18
86	Q96QD8	SLC38A2	Sodium-coupled neutral amino acid transporter 2 OS = Homo sapiens OX = 9606 GN = SLC38A2 PE = 1 SV = 2	3.85 × 10^8^	3.36 × 10^9^	−3.125	18
87	Q99523	SORT1	Sortilin OS = Homo sapiens OX = 9606 GN = SORT1 PE = 1 SV = 3	3.49 × 10^8^	4.82 × 10^8^	−0.466	18
88	Q9C0H2-4	TTYH3	Isoform 4 of Protein tweety homolog 3 OS = Homo sapiens OX = 9606 GN = TTYH3	2.51 × 10^8^	4.19 × 10^8^	−0.739	18
89	Q9HD45	TM9SF3	Transmembrane 9 superfamily member 3 OS = Homo sapiens OX = 9606 GN = TM9SF3 PE = 1 SV = 2	3.88 × 10^8^	4.46 × 10^8^	−0.201	18
90	Q9Y3B3	TMED7	Transmembrane emp24 domain-containing protein 7 OS = Homo sapiens OX = 9606 GN = TMED7 PE = 1 SV = 2	2.92 × 10^8^	3.64 × 10^8^	−0.318	18
91	Q9Y487	ATP6V0A2	V-type proton ATPase 116 kDa subunit a2 OS = Homo sapiens OX = 9606 GN = ATP6V0A2 PE = 1 SV = 2	6.50 × 10^7^	4.24 × 10^7^	0.617	18
92	Q9Y624	F11R	Junctional adhesion molecule A OS = Homo sapiens OX = 9606 GN = F11R PE = 1 SV = 1; Isoform 2 of Junctional adhesion molecule A OS = Homo sapiens OX = 9606 GN = F11R	9.98 × 10^6^	0.00	10.000	18
93	A8MWY0	ELAPOR2	Endosome/lysosome-associated apoptosis and autophagy regulator family member 2 OS = Homo sapiens OX = 9606 GN = ELAPOR2 PE = 1 SV = 2	6.32 × 10^6^	0.00	10.000	18.5
94	P19022-2	CDH2	Isoform 2 of Cadherin-2 OS = Homo sapiens OX = 9606 GN = CDH2; Cadherin-2 OS = Homo sapiens OX = 9606 GN = CDH2 PE = 1 SV = 4	1.17 × 10^8^	6.33 × 10^7^	0.888	18.5
95	Q13641	TPBG	Trophoblast glycoprotein OS = Homo sapiens OX = 9606 GN = TPBG PE = 1 SV = 1	2.32 × 10^8^	3.40 × 10^8^	−0.555	18.5
96	Q8N158	GPC2	Glypican-2 OS = Homo sapiens OX = 9606 GN = GPC2 PE = 2 SV = 1	6.34 × 10^6^	0.00	10.000	18.5
97	Q99758	ABCA3	Phospholipid-transporting ATPase ABCA3 OS = Homo sapiens OX = 9606 GN = ABCA3 PE = 1 SV = 2	2.36 × 10^7^	0.00	10.000	18.5
98	Q9HCN3	PGAP6	Post-GPI attachment to proteins factor 6 OS = Homo sapiens OX = 9606 GN = PGAP6 PE = 1 SV = 3	7.34 × 10^6^	0.00	10.000	18.5
99	O14672	ADAM10	Disintegrin and metalloproteinase domain-containing protein 10 OS = Homo sapiens OX = 9606 GN = ADAM10 PE = 1 SV = 1	1.34 × 10^8^	3.71 × 10^8^	−1.474	19
100	O15118	NPC1	NPC intracellular cholesterol transporter 1 OS = Homo sapiens OX = 9606 GN = NPC1 PE = 1 SV = 2	2.08 × 10^8^	1.87 × 10^8^	0.148	19

## References

[1] Hawkins D.S., Spunt S.L., Skapek S.X., COG Soft Tissue Sarcoma Committee (2013). Children’s Oncology Group’s 2013 blueprint for research: Soft tissue sarcomas. Pediatr. Blood Cancer.

[2] Siegel R.L., Miller K.D., Fuchs H.E., Jemal A. (2022). Cancer statistics, 2022. CA Cancer J. Clin..

[3] Hettmer S., Wagers A.J. (2010). Muscling in: Uncovering the origins of rhabdomyosarcoma. Nat. Med..

[4] The WHO Classification of Tumours Editorial Board (2020). WHO Classification of Tumours Soft Tissue and Bone Tumours.

[5] Barr F.G., Smith L.M., Lynch J.C., Strzelecki D., Parham D.M., Qualman S.J., Breitfeld P.P. (2006). Examination of gene fusion status in archival samples of alveolar rhabdomyosarcoma entered on the Intergroup Rhabdomyosarcoma Study-III trial: A report from the Children’s Oncology Group. J. Mol. Diagn..

[6] Williamson D., Missiaglia E., de Reynies A., Pierron G., Thuille B., Palenzuela G., Thway K., Orbach D., Lae M., Freneaux P. (2010). Fusion gene-negative alveolar rhabdomyosarcoma is clinically and molecularly indistinguishable from embryonal rhabdomyosarcoma. J. Clin. Oncol..

[7] Malempati S., Weigel B.J., Chi Y.Y., Tian J., Anderson J.R., Parham D.M., Teot L.A., Rodeberg D.A., Yock T.I., Shulkin B.L. (2019). The addition of cixutumumab or temozolomide to intensive multiagent chemotherapy is feasible but does not improve outcome for patients with metastatic rhabdomyosarcoma: A report from the Children’s Oncology Group. Cancer.

[8] Shern J.F., Selfe J., Izquierdo E., Patidar R., Chou H.C., Song Y.K., Yohe M.E., Sindiri S., Wei J., Wen X. (2021). Genomic Classification and Clinical Outcome in Rhabdomyosarcoma: A Report From an International Consortium. J. Clin. Oncol..

[9] Punyko J.A., Mertens A.C., Gurney J.G., Yasui Y., Donaldson S.S., Rodeberg D.A., Raney R.B., Stovall M., Sklar C.A., Robison L.L. (2005). Long-term medical effects of childhood and adolescent rhabdomyosarcoma: A report from the childhood cancer survivor study. Pediatr. Blood Cancer.

[10] Owosho A.A., Brady P., Wolden S.L., Wexler L.H., Antonescu C.R., Huryn J.M., Estilo C.L. (2016). Long-term effect of chemotherapy-intensity-modulated radiation therapy (chemo-IMRT) on dentofacial development in head and neck rhabdomyosarcoma patients. Pediatr. Hematol. Oncol..

[11] Zolot R.S., Basu S., Million R.P. (2013). Antibody-drug conjugates. Nat. Rev. Drug Discov..

[12] Drago J.Z., Modi S., Chandarlapaty S. (2021). Unlocking the potential of antibody-drug conjugates for cancer therapy. Nat. Rev. Clin. Oncol..

[13] Blanco B., Dominguez-Alonso C., Alvarez-Vallina L. (2021). Bispecific Immunomodulatory Antibodies for Cancer Immunotherapy. Clin. Cancer Res..

[14] Esfandiari A., Cassidy S., Webster R.M. (2022). Bispecific antibodies in oncology. Nat. Rev. Drug Discov..

[15] June C.H., Sadelain M. (2018). Chimeric Antigen Receptor Therapy. N. Engl. J. Med..

[16] Larson R.C., Maus M.V. (2021). Recent advances and discoveries in the mechanisms and functions of CAR T cells. Nat. Rev. Cancer.

[17] June C.H., O’Connor R.S., Kawalekar O.U., Ghassemi S., Milone M.C. (2018). CAR T cell immunotherapy for human cancer. Science.

[18] El-Badry O.M., Minniti C., Kohn E.C., Houghton P.J., Daughaday W.H., Helman L.J. (1990). Insulin-like growth factor II acts as an autocrine growth and motility factor in human rhabdomyosarcoma tumors. Cell Growth Differ..

[19] Wan X., Helman L.J. (2003). Levels of PTEN protein modulate Akt phosphorylation on serine 473, but not on threonine 308, in IGF-II-overexpressing rhabdomyosarcomas cells. Oncogene.

[20] Shapiro D.N., Jones B.G., Shapiro L.H., Dias P., Houghton P.J. (1994). Antisense-mediated reduction in insulin-like growth factor-I receptor expression suppresses the malignant phenotype of a human alveolar rhabdomyosarcoma. J. Clin. Investig..

[21] Gattenloehner S., Vincent A., Leuschner I., Tzartos S., Muller-Hermelink H.K., Kirchner T., Marx A. (1998). The fetal form of the acetylcholine receptor distinguishes rhabdomyosarcomas from other childhood tumors. Am. J. Pathol..

[22] Mechtersheimer G., Staudter M., Möller P. (1991). Expression of the Natural Killer Cell-associated Antigens CD56 and CD57 in Human Neural and Striated Muscle Cells and in Their Tumors1. Cancer Res..

[23] Phimister E.G., Culverwell A., Patel K., Kemshead J.T. (1994). Tissue-specific expression of neural cell adhesion molecule (NCAM) may allow differential diagnosis of neuroblastoma from embryonal rhabdomyosarcoma. Eur. J. Cancer.

[24] Gluer S., Schelp C., von Schweinitz D., Gerardy-Schahn R. (1998). Polysialylated neural cell adhesion molecule in childhood rhabdomyosarcoma. Pediatr. Res..

[25] Bahrami A., Gown A.M., Baird G.S., Hicks M.J., Folpe A.L. (2008). Aberrant expression of epithelial and neuroendocrine markers in alveolar rhabdomyosarcoma: A potentially serious diagnostic pitfall. Mod. Pathol..

[26] De Giovanni C., Landuzzi L., Frabetti F., Nicoletti G., Griffoni C., Rossi I., Mazzotti M., Scotto L., Nanni P., Lollini P.-L. (1996). Antisense Epidermal Growth Factor Receptor Transfection Impairs the Proliferative Ability of Human Rhabdomyosarcoma Cells. Cancer Res..

[27] Andrechek E.R., Hardy W.R., Girgis-Gabardo A.A., Perry R.L., Butler R., Graham F.L., Kahn R.C., Rudnicki M.A., Muller W.J. (2002). ErbB2 is required for muscle spindle and myoblast cell survival. Mol. Cell. Biol..

[28] Nanni P., Nicoletti G., De Giovanni C., Croci S., Astolfi A., Landuzzi L., Di Carlo E., Iezzi M., Musiani P., Lollini P.-L. (2003). Development of Rhabdomyosarcoma in HER-2/neu Transgenic p53 Mutant Mice1. Cancer Res..

[29] Armistead P.M., Salganick J., Roh J.S., Steinert D.M., Patel S., Munsell M., El-Naggar A.K., Benjamin R.S., Zhang W., Trent J.C. (2007). Expression of receptor tyrosine kinases and apoptotic molecules in rhabdomyosarcoma: Correlation with overall survival in 105 patients. Cancer.

[30] Ganti R., Skapek S.X., Zhang J., Fuller C.E., Wu J., Billups C.A., Breitfeld P.P., Dalton J.D., Meyer W.H., Khoury J.D. (2006). Expression and genomic status of EGFR and ErbB-2 in alveolar and embryonal rhabdomyosarcoma. Mod. Pathol..

[31] Mayeenuddin L.H., Yu Y., Kang Z., Helman L.J., Cao L. (2010). Insulin-like growth factor 1 receptor antibody induces rhabdomyosarcoma cell death via a process involving AKT and Bcl-x(L). Oncogene.

[32] Kalebic T., Tsokos M., Helman L.J. (1994). In Vivo Treatment with Antibody against IGF-1 Receptor Suppresses Growth of Human Rhabdomyosarcoma and Down-Regulates p34cdc2. Cancer Res..

[33] Gombos A., Metzger-Filho O., Dal Lago L., Awada-Hussein A. (2012). Clinical development of insulin-like growth factor receptor--1 (IGF-1R) inhibitors: At the crossroad?. Investig. New Drugs.

[34] Simon-Keller K., Barth S., Vincent A., Marx A. (2013). Targeting the fetal acetylcholine receptor in rhabdomyosarcoma. Expert. Opin. Targets.

[35] Geller J.I., Pressey J.G., Smith M.A., Kudgus R.A., Cajaiba M., Reid J.M., Hall D., Barkauskas D.A., Voss S.D., Cho S.Y. (2020). ADVL1522: A phase 2 study of lorvotuzumab mertansine (IMGN901) in children with relapsed or refractory wilms tumor, rhabdomyosarcoma, neuroblastoma, pleuropulmonary blastoma, malignant peripheral nerve sheath tumor, or synovial sarcoma-A Children’s Oncology Group study. Cancer.

[36] Hegde M., Joseph S.K., Pashankar F., DeRenzo C., Sanber K., Navai S., Byrd T.T., Hicks J., Xu M.L., Gerken C. (2020). Tumor response and endogenous immune reactivity after administration of HER2 CAR T cells in a child with metastatic rhabdomyosarcoma. Nat. Commun..

[37] Khan J., Wei J.S., Ringner M., Saal L.H., Ladanyi M., Westermann F., Berthold F., Schwab M., Antonescu C.R., Peterson C. (2001). Classification and diagnostic prediction of cancers using gene expression profiling and artificial neural networks. Nat. Med..

[38] Baird K., Davis S., Antonescu C.R., Harper U.L., Walker R.L., Chen Y., Glatfelter A.A., Duray P.H., Meltzer P.S. (2005). Gene expression profiling of human sarcomas: Insights into sarcoma biology. Cancer Res..

[39] Davicioni E., Finckenstein F.G., Shahbazian V., Buckley J.D., Triche T.J., Anderson M.J. (2006). Identification of a PAX-FKHR gene expression signature that defines molecular classes and determines the prognosis of alveolar rhabdomyosarcomas. Cancer Res..

[40] Baskar S., Shivaprasad N., Zhu Z., Dimitrov D., Sigrist M., Sorensen P., Yohe M., Shern J., Maris J., Mackall C. (2015). Abstract 2488: FGFR4 as a potential therapeutic target for monoclonal antibody based intervention in rhabdomyosarcoma. Cancer Res..

[41] Meyer M.J., Jenkins D., Batt D., Mosher R., Isaacs R., Hu T., Capka V., Zhang X., Chen D., Tang L. (2015). Abstract 1680: In vitro and in vivo activity of a highly potent and novel FGFR2/FGFR4 dual targeting antibody-drug conjugate. Cancer Res..

[42] Sullivan P.M., Kumar R., Li W., Hoglund V., Wang L., Zhang Y., Shi M., Baek D., Cheuk A., Jensen M.C. (2022). FGFR4-targeted chimeric antigen receptors (CARs) combined with anti-myeloid poly-pharmacy effectively treats orthotopic rhabdomyosarcoma. Mol. Cancer Ther..

[43] Alijaj N., Moutel S., Gouveia Z.L., Gray M., Roveri M., Dzhumashev D., Weber F., Meier G., Luciani P., Rössler J.K. (2020). Novel FGFR4-Targeting Single-Domain Antibodies for Multiple Targeted Therapies against Rhabdomyosarcoma. Cancers.

[44] Oesch S., Walter D., Wachtel M., Pretre K., Salazar M., Guzman M., Velasco G., Schafer B.W. (2009). Cannabinoid receptor 1 is a potential drug target for treatment of translocation-positive rhabdomyosarcoma. Mol. Cancer Ther..

[45] Orentas R.J., Yang J.J., Wen X., Wei J.S., Mackall C.L., Khan J. (2012). Identification of cell surface proteins as potential immunotherapy targets in 12 pediatric cancers. Front. Oncol..

[46] Orentas R.J., Lee D.W., Mackall C. (2012). Immunotherapy targets in pediatric cancer. Front. Oncol..

[47] Shern J.F., Chen L., Chmielecki J., Wei J.S., Patidar R., Rosenberg M., Ambrogio L., Auclair D., Wang J., Song Y.K. (2014). Comprehensive genomic analysis of rhabdomyosarcoma reveals a landscape of alterations affecting a common genetic axis in fusion-positive and fusion-negative tumors. Cancer Discov..

[48] Stewart E., McEvoy J., Wang H., Chen X., Honnell V., Ocarz M., Gordon B., Dapper J., Blankenship K., Yang Y. (2018). Identification of Therapeutic Targets in Rhabdomyosarcoma through Integrated Genomic, Epigenomic, and Proteomic Analyses. Cancer Cell.

[49] Brohl A.S., Sindiri S., Wei J.S., Milewski D., Chou H.C., Song Y.K., Wen X., Kumar J., Reardon H.V., Mudunuri U.S. (2021). Immuno-transcriptomic profiling of extracranial pediatric solid malignancies. Cell Rep..

[50] Vogel C., Marcotte E.M. (2012). Insights into the regulation of protein abundance from proteomic and transcriptomic analyses. Nat. Rev. Genet..

[51] Lavoie R.R., Gargollo P.C., Ahmed M.E., Kim Y., Baer E., Phelps D.A., Charlesworth C.M., Madden B.J., Wang L., Houghton P.J. (2021). Surfaceome Profiling of Rhabdomyosarcoma Reveals B7-H3 as a Mediator of Immune Evasion. Cancers.

[52] Lin P.H., Selinfreund R., Wakshull E., Wharton W. (1987). Rapid and efficient purification of plasma membrane from cultured cells: Characterization of epidermal growth factor binding. Biochemistry.

[53] Bausch-Fluck D., Goldmann U., Muller S., van Oostrum M., Muller M., Schubert O.T., Wollscheid B. (2018). The in silico human surfaceome. Proc. Natl. Acad. Sci. USA.

[54] Ritchie M.E., Phipson B., Wu D., Hu Y., Law C.W., Shi W., Smyth G.K. (2015). Limma powers differential expression analyses for RNA-sequencing and microarray studies. Nucleic Acids Res..

[55] Kammers K., Cole R.N., Tiengwe C., Ruczinski I. (2015). Detecting Significant Changes in Protein Abundance. EuPA Open Proteom..

[56] Hoffman G.E., Roussos P. (2021). Dream: Powerful differential expression analysis for repeated measures designs. Bioinformatics.

[57] Schmidt T., Samaras P., Frejno M., Gessulat S., Barnert M., Kienegger H., Krcmar H., Schlegl J., Ehrlich H.C., Aiche S. (2018). ProteomicsDB. Nucleic. Acids. Res..

[58] Samaras P., Schmidt T., Frejno M., Gessulat S., Reinecke M., Jarzab A., Zecha J., Mergner J., Giansanti P., Ehrlich H.C. (2020). ProteomicsDB: A multi-omics and multi-organism resource for life science research. Nucleic Acids Res..

[59] Lautenbacher L., Samaras P., Muller J., Grafberger A., Shraideh M., Rank J., Fuchs S.T., Schmidt T.K., The M., Dallago C. (2022). ProteomicsDB: Toward a FAIR open-source resource for life-science research. Nucleic Acids Res..

[60] Azorsa D.O., Bode P.K., Wachtel M., Cheuk A.T.C., Meltzer P.S., Vokuhl C., Camenisch U., Khov H.L., Bode B., Schafer B.W. (2021). Immunohistochemical detection of PAX-FOXO1 fusion proteins in alveolar rhabdomyosarcoma using breakpoint specific monoclonal antibodies. Mod. Pathol..

[61] Cox D.R. (1972). Regression Models and Life-Tables. J. R. Stat. Soc. Ser. B Methodol..

[62] Majzner R.G., Theruvath J.L., Nellan A., Heitzeneder S., Cui Y., Mount C.W., Rietberg S.P., Linde M.H., Xu P., Rota C. (2019). CAR T cells targeting B7-H3, a pan-cancer antigen, demonstrate potent preclinical activity against pediatric solid tumors and brain tumors. Clin. Cancer Res..

[63] Kanayama T., Miyachi M., Sugimoto Y., Yagyu S., Kikuchi K., Tsuchiya K., Iehara T., Hosoi H. (2021). Reduced B7-H3 expression by PAX3-FOXO1 knockdown inhibits cellular motility and promotes myogenic differentiation in alveolar rhabdomyosarcoma. Sci. Rep..

[64] Modak S., Kramer K., Gultekin S.H., Guo H.F., Cheung N.K. (2001). Monoclonal antibody 8H9 targets a novel cell surface antigen expressed by a wide spectrum of human solid tumors. Cancer Res..

[65] Modak S., Guo H.F., Humm J.L., Smith-Jones P.M., Larson S.M., Cheung N.K. (2005). Radioimmunotargeting of human rhabdomyosarcoma using monoclonal antibody 8H9. Cancer Biother. Radiopharm..

[66] Kendsersky N.M., Lindsay J., Kolb E.A., Smith M.A., Teicher B.A., Erickson S.W., Earley E.J., Mosse Y.P., Martinez D., Pogoriler J. (2021). The B7-H3-Targeting Antibody-Drug Conjugate m276-SL-PBD Is Potently Effective Against Pediatric Cancer Preclinical Solid Tumor Models. Clin. Cancer Res..

[67] Meli M.L., Carrel F., Waibel R., Amstutz H., Crompton N., Jaussi R., Moch H., Schubiger P.A., Novak-Hofer I. (1999). Anti-neuroblastoma antibody chCE7 binds to an isoform of L1-CAM present in renal carcinoma cells. Int. J. Cancer.

[68] Kunkele A., Johnson A.J., Rolczynski L.S., Chang C.A., Hoglund V., Kelly-Spratt K.S., Jensen M.C. (2015). Functional Tuning of CARs Reveals Signaling Threshold above Which CD8+ CTL Antitumor Potency Is Attenuated due to Cell Fas-FasL-Dependent AICD. Cancer Immunol. Res..

[69] Kunkele A., Taraseviciute A., Finn L.S., Johnson A.J., Berger C., Finney O., Chang C.A., Rolczynski L.S., Brown C., Mgebroff S. (2017). Preclinical Assessment of CD171-Directed CAR T-cell Adoptive Therapy for Childhood Neuroblastoma: CE7 Epitope Target Safety and Product Manufacturing Feasibility. Clin. Cancer Res..

[70] Hong H., Stastny M., Brown C., Chang W.C., Ostberg J.R., Forman S.J., Jensen M.C. (2014). Diverse solid tumors expressing a restricted epitope of L1-CAM can be targeted by chimeric antigen receptor redirected T lymphocytes. J. Immunother..

[71] Daponte A., Kostopoulou E., Kollia P., Papamichali R., Vanakara P., Hadjichristodoulou C., Nakou M., Samara S., Koukoulis G., Messinis I.E. (2008). L1 (CAM) (CD171) in ovarian serous neoplasms. Eur. J. Gynaecol. Oncol..

[72] Fankhauser C.D., Bode P.K., Hermanns T., Sander S., Beyer J., Sulser T., Altevogt P., Moch H., Tischler V. (2016). L1-CAM is commonly expressed in testicular germ cell tumours. J. Clin. Pathol..

[73] Park J.R., Digiusto D.L., Slovak M., Wright C., Naranjo A., Wagner J., Meechoovet H.B., Bautista C., Chang W.C., Ostberg J.R. (2007). Adoptive transfer of chimeric antigen receptor re-directed cytolytic T lymphocyte clones in patients with neuroblastoma. Mol. Ther..

[74] Inaguma S., Wang Z., Lasota J.P., Miettinen M.M. (2016). Expression of neural cell adhesion molecule L1 (CD171) in neuroectodermal and other tumors: An immunohistochemical study of 5155 tumors and critical evaluation of CD171 prognostic value in gastrointestinal stromal tumors. Oncotarget.

[75] White G.R., Varley J.M., Heighway J. (2000). Genomic structure and expression profile of LPHH1, a 7TM gene variably expressed in breast cancer cell lines. Biochim. Biophys. Acta.

[76] Bondarev A.D., Attwood M.M., Jonsson J., Chubarev V.N., Tarasov V.V., Schioth H.B. (2020). Opportunities and challenges for drug discovery in modulating Adhesion G protein-coupled receptor (GPCR) functions. Expert Opin. Drug Discov..

[77] Zhang S., Liu Y., Liu Z., Zhang C., Cao H., Ye Y., Wang S., Zhang Y., Xiao S., Yang P. (2014). Transcriptome profiling of a multiple recurrent muscle-invasive urothelial carcinoma of the bladder by deep sequencing. PLoS ONE.

[78] Pellissier F., Gerber A., Bauer C., Ballivet M., Ossipow V. (2007). The adhesion molecule Necl-3/SynCAM-2 localizes to myelinated axons, binds to oligodendrocytes and promotes cell adhesion. BMC Neurosci..

[79] Rathjen T., Yan X., Kononenko N.L., Ku M.C., Song K., Ferrarese L., Tarallo V., Puchkov D., Kochlamazashvili G., Brachs S. (2017). Regulation of body weight and energy homeostasis by neuronal cell adhesion molecule 1. Nat. Neurosci..

[80] Liu N., Yang C., Bai W., Wang Z., Wang X., Johnson M., Wang W., Zhang P., Yang H., Liu H. (2019). CADM2 inhibits human glioma proliferation, migration and invasion. Oncol. Rep..

[81] Chang G., Xu S., Dhir R., Chandran U., O’Keefe D.S., Greenberg N.M., Gingrich J.R. (2010). Hypoexpression and epigenetic regulation of candidate tumor suppressor gene CADM-2 in human prostate cancer. Clin. Cancer Res..

[82] He W., Li X., Xu S., Ai J., Gong Y., Gregg J.L., Guan R., Qiu W., Xin D., Gingrich J.R. (2013). Aberrant methylation and loss of CADM2 tumor suppressor expression is associated with human renal cell carcinoma tumor progression. Biochem. Biophys. Res. Commun..

[83] Dai L., Zhao J., Yin J., Fu W., Chen G. (2020). Cell adhesion molecule 2 (CADM2) promotes brain metastasis by inducing epithelial-mesenchymal transition (EMT) in human non-small cell lung cancer. Ann. Transl. Med..

[84] Li D., Zhang Y., Zhang H., Zhan C., Li X., Ba T., Qiu Z., Fang E., Lv G., Zou C. (2018). CADM2, as a new target of miR-10b, promotes tumor metastasis through FAK/AKT pathway in hepatocellular carcinoma. J. Exp. Clin. Cancer Res..

[85] Chung W.S., Clarke L.E., Wang G.X., Stafford B.K., Sher A., Chakraborty C., Joung J., Foo L.C., Thompson A., Chen C. (2013). Astrocytes mediate synapse elimination through MEGF10 and MERTK pathways. Nature.

[86] Seale P., Ishibashi J., Holterman C., Rudnicki M.A. (2004). Muscle satellite cell-specific genes identified by genetic profiling of MyoD-deficient myogenic cell. Dev. Biol..

[87] Saha M., Mitsuhashi S., Jones M.D., Manko K., Reddy H.M., Bruels C.C., Cho K.A., Pacak C.A., Draper I., Kang P.B. (2017). Consequences of MEGF10 deficiency on myoblast function and Notch1 interactions. Hum. Mol. Genet..

[88] Ganassi M., Muntoni F., Zammit P.S. (2022). Defining and identifying satellite cell-opathies within muscular dystrophies and myopathies. Exp. Cell Res..

[89] Lak N.S.M., Voormanns T.L., Zappeij-Kannegieter L., van Zogchel L.M.J., Fiocco M., van Noesel M.M., Merks J.H.M., van der Schoot C.E., Tytgat G.A.M., Stutterheim J. (2021). Improving Risk Stratification for Pediatric Patients with Rhabdomyosarcoma by Molecular Detection of Disseminated Disease. Clin. Cancer Res..

[90] Holterman C.E., Le Grand F., Kuang S., Seale P., Rudnicki M.A. (2007). Megf10 regulates the progression of the satellite cell myogenic program. J. Cell. Biol..

[91] Williamson D., Selfe J., Gordon T., Lu Y.J., Pritchard-Jones K., Murai K., Jones P., Workman P., Shipley J. (2007). Role for amplification and expression of glypican-5 in rhabdomyosarcoma. Cancer Res..

[92] Li F., Shi W., Capurro M., Filmus J. (2011). Glypican-5 stimulates rhabdomyosarcoma cell proliferation by activating Hedgehog signaling. J. Cell Biol..

[93] Li N., Spetz M.R., Ho M. (2020). The Role of Glypicans in Cancer Progression and Therapy. J. Histochem. Cytochem..

[94] Liang T.W., Chiu H.H., Gurney A., Sidle A., Tumas D.B., Schow P., Foster J., Klassen T., Dennis K., DeMarco R.A. (2002). Vascular endothelial-junctional adhesion molecule (VE-JAM)/JAM 2 interacts with T, NK, and dendritic cells through JAM 3. J. Immunol..

[95] Arrate M.P., Rodriguez J.M., Tran T.M., Brock T.A., Cunningham S.A. (2001). Cloning of human junctional adhesion molecule 3 (JAM3) and its identification as the JAM2 counter-receptor. J. Biol. Chem..

[96] Powell G.T., Wright G.J. (2011). Jamb and jamc are essential for vertebrate myocyte fusion. PLoS Biol..

[97] Hromowyk K.J., Talbot J.C., Martin B.L., Janssen P.M.L., Amacher S.L. (2020). Cell fusion is differentially regulated in zebrafish post-embryonic slow and fast muscle. Dev. Biol..

[98] Lauko A., Mu Z., Gutmann D.H., Naik U.P., Lathia J.D. (2020). Junctional Adhesion Molecules in Cancer: A Paradigm for the Diverse Functions of Cell-Cell Interactions in Tumor Progression. Cancer Res..

[99] Hosonaga M., Arima Y., Sampetrean O., Komura D., Koya I., Sasaki T., Sato E., Okano H., Kudoh J., Ishikawa S. (2018). HER2 Heterogeneity Is Associated with Poor Survival in HER2-Positive Breast Cancer. Int. J. Mol. Sci..

[100] Manzella G., Schreck L.D., Breunis W.B., Molenaar J., Merks H., Barr F.G., Sun W., Rommele M., Zhang L., Tchinda J. (2020). Phenotypic profiling with a living biobank of primary rhabdomyosarcoma unravels disease heterogeneity and AKT sensitivity. Nat. Commun..

[101] Danielli S.G., Porpiglia E., De Micheli A.J., Navarro N., Zellinger M.J., Bechtold I., Kisele S., Volken L., Marques J.G., Kasper S. (2022). Single-cell mapping of tumor heterogeneity in pediatric rhabdomyosarcoma reveals developmental signatures with therapeutic relevance. BioRxiv.

[102] Gunasekera K., Wuthrich D., Braga-Lagache S., Heller M., Ochsenreiter T. (2012). Proteome remodelling during development from blood to insect-form Trypanosoma brucei quantified by SILAC and mass spectrometry. BMC Genom..

[103] Cox J., Mann M. (2008). MaxQuant enables high peptide identification rates, individualized p.p.b.-range mass accuracies and proteome-wide protein quantification. Nat. Biotechnol..

[104] UniProt C. (2019). UniProt: A worldwide hub of protein knowledge. Nucleic Acids Res..

[105] Huber W., von Heydebreck A., Sultmann H., Poustka A., Vingron M. (2002). Variance stabilization applied to microarray data calibration and to the quantification of differential expression. Bioinformatics.

[106] Silver J.D., Ritchie M.E., Smyth G.K. (2009). Microarray background correction: Maximum likelihood estimation for the normal-exponential convolution. Biostatistics.

[107] Lee J.K., Bangayan N.J., Chai T., Smith B.A., Pariva T.E., Yun S., Vashisht A., Zhang Q., Park J.W., Corey E. (2018). Systemic surfaceome profiling identifies target antigens for immune-based therapy in subtypes of advanced prostate cancer. Proc. Natl. Acad. Sci. USA.

[108] Ashburner M., Ball C.A., Blake J.A., Botstein D., Butler H., Cherry J.M., Davis A.P., Dolinski K., Dwight S.S., Eppig J.T. (2000). Gene ontology: Tool for the unification of biology. The Gene Ontology Consortium. Nat. Genet..

[109] Sonnhammer E.L., von Heijne G., Krogh A. (1998). A hidden Markov model for predicting transmembrane helices in protein sequences. Proc. Int. Conf. Intell. Syst. Mol. Biol..

[110] Uldry A.C., Maciel-Dominguez A., Jornod M., Buchs N., Braga-Lagache S., Brodard J., Jankovic J., Bonadies N., Heller M. (2022). Effect of Sample Transportation on the Proteome of Human Circulating Blood Extracellular Vesicles. Int. J. Mol. Sci..

[111] Goedhart J., Luijsterburg M.S. (2020). VolcaNoseR is a web app for creating, exploring, labeling and sharing volcano plots. Sci. Rep..

[112] Davicioni E., Anderson M.J., Finckenstein F.G., Lynch J.C., Qualman S.J., Shimada H., Schofield D.E., Buckley J.D., Meyer W.H., Sorensen P.H. (2009). Molecular classification of rhabdomyosarcoma—Genotypic and phenotypic determinants of diagnosis: A report from the Children’s Oncology Group. Am. J. Pathol..

[113] Lanczky A., Gyorffy B. (2021). Web-Based Survival Analysis Tool Tailored for Medical Research (KMplot): Development and Implementation. J. Med. Internet Res..

[114] Deutsch E.W., Bandeira N., Sharma V., Perez-Riverol Y., Carver J.J., Kundu D.J., Garcia-Seisdedos D., Jarnuczak A.F., Hewapathirana S., Pullman B.S. (2020). The ProteomeXchange consortium in 2020: Enabling ‘big data’ approaches in proteomics. Nucleic Acids Res..

